# Interaction of the Antibiotic Rifampicin with Lipid Membranes

**DOI:** 10.3390/biom15030320

**Published:** 2025-02-21

**Authors:** Rui M. S. Santos, Jaime Samelo, Alexandre C. Oliveira, Margarida M. Cordeiro, Maria Julia Mora, Gladys E. Granero, Hugo A. L. Filipe, Luís M. S. Loura, Maria João Moreno

**Affiliations:** 1 Coimbra Chemistry Center, Institute of Molecular Sciences (CQC-IMS), University of Coimbra, 3004-535 Coimbra, Portugalac_oliveira10@hotmail.com (A.C.O.);; 2 Department of Chemistry, Faculty of Sciences and Technology, University of Coimbra, 3004-535 Coimbra, Portugal; 3 Unidad de Investigación y Desarrollo en Tecnología Farmacéutica (UNITEFA, CONICET) and Departamento de Ciencias Farmacéuticas, Facultad de Ciencias Químicas, Universidad Nacional de Córdoba, Ciudad Universitaria, Córdoba 5000, Argentina; mjmora@unc.edu.ar (M.J.M.); glagranero@unc.edu.ar (G.E.G.); 4 BRIDGES-Biotechnology Research, Innovation, and Design of Health Products, Polytechnic of Guarda, Av. Dr. Francisco Sá Carneiro, 50, 6300-559 Guarda, Portugal; hlfilipe@ipg.pt; 5 CNC—Center for Neuroscience and Cell Biology, University of Coimbra, 3004-535 Coimbra, Portugal; 6 Faculty of Pharmacy, University of Coimbra, 3000-548 Coimbra, Portugal

**Keywords:** drug–membrane association, membrane perturbation, drug bioavailability

## Abstract

Rifampicin is a broad-spectrum antibiotic, active against several bacterial infections such as tuberculosis. It is a relatively large and structurally complex molecule, including numerous polar groups. Although violating several of Lipinski’s rules for efficient intestinal absorption, rifampicin shows good oral bioavailability, permeating through cell membranes in the absorption pathway and those of the target organisms. Some hypotheses have been proposed for its efficient membrane permeation, but the details are mostly unknown. In this work, the interaction of rifampicin with POPC lipid bilayers is studied using experimental biophysics methodologies and atomistic molecular dynamics simulations considering the two most prevalent ionic species at physiological pH, the anionic and the zwitterionic forms. The results show that both ionization forms of rifampicin establish favorable interactions with the membrane lipids, in agreement with the relatively high partition coefficient obtained experimentally. The results from MD simulations and isothermal titration calorimetry using different pH buffers show that the piperazine group inserts deeply in the membrane and is accompanied by a stabilization of its neutral form. The bulky nature of rifampicin and its deep insertion in the membrane lead to a strong perturbation in the lipids local order, decreasing the membrane barrier properties as evaluated from the rate of carboxyfluorescein leaching. Altogether, the comparison between the experimental and MD simulations results provides important insight regarding the rifampicin molecular features responsible for its relatively fast membrane permeation. The lipid POPC used in this study was selected as a simple membrane with relevance for different organisms across all kingdoms. Further studies using more complex lipid compositions will provide details on eventual specificities for rifampicin interaction with the membranes of distinct organisms.

## 1. Introduction

The interactions between drugs and biological membranes play a crucial role in determining drug pharmacokinetics and therapeutic efficacy. Membranes act as selective barriers, influencing the absorption, distribution, metabolism, and excretion of drugs. The extent to which a drug can permeate cell membranes affects its bioavailability and overall effectiveness. Drugs that interact favorably with the lipid bilayer are more likely to cross biological membranes efficiently, impacting their concentration at target sites [1]. Therefore, understanding how drug molecules engage with these membranes is essential for predicting their behavior in the body, including their ability to reach and act on their intended targets. Furthermore, optimizing these interactions is key for enhancing a drug’s therapeutic potential and minimizing side effects.

In recent years, there has been a notable trend toward increasing the molecular weight of drug candidates to improve specificity for their molecular targets [2,3]. Larger molecules often exhibit enhanced target selectivity, potentially leading to higher therapeutic efficacy and fewer off-target effects. However, this increase in molecular weight presents challenges, particularly regarding oral bioavailability. According to Lipinski’s rule of five, drugs with a molecular weight above 500 g/mol are more prone to show reduced membrane permeability and/or solubility, making it harder for them to be absorbed into the bloodstream when administered orally [4]. Despite this, many drugs with molecular weights exceeding this threshold and breaking several of Lipinski’s rules still show high bioavailability and efficacy (drugs beyond rules of 5, bRO5) [2,3,5,6,7,8]. By investigating how such drugs achieve effective membrane permeation and target interaction, researchers can unlock new strategies for designing high-molecular-weight drugs with improved oral bioavailability, guiding the future development of therapeutics that break traditional boundaries.

Rifampicin is an example of a bRO5 drug, highly effective and with notable oral bioavailability. With a molecular weight over 800 g/mol and a complex structure including 6 donor and 25 acceptor sites able to participate in H bonds at neutral pH values [9], above the limits established by Lipinski’s rule (5 and 10, respectively), the antibiotic rifampicin challenges conventional expectations showing an oral bioavailability of 90 to 95% [10]. Some studies have sought to rationalize this behavior, highlighting its “molecular chameleon” behavior with the ability to form intramolecular hydrogen bonds [6,7,8,11]. This decreases the enthalpic penalty upon partition into non-protic media, as well as the polar surface area exposed to the non-polar medium, thus facilitating its permeation through the non-polar core of biomembranes and increasing its bioavailability. Another distinctive aspect of the rifampicin molecular structure is the presence of both weak acid and weak base groups, the phenolic and the piperazine moieties. When in aqueous media, the first ionization of the phenolic groups occurs at acidic pH values, while piperazine deprotonates for slightly alkaline pH values (reported p*K*_a_ values of 3 and 7.5, and 1.7 and 7.9 in references [7,12], respectively). The zwitterionic species is, thus, the most abundant at neutral pH values, with significant fractions of the negatively charged species. The stability of the neutral form of both ionizing moieties increases when the medium polarity decreases, leading to an increase in the p*K*_a_ of the phenolic groups and a decrease in that of piperazine [7,12]. A decrease in the relative abundance of the zwitterionic over the neutral form has in fact been observed by spectrophotometry when the solvent polarity is decreased [7], allowing to rationalize rifampicin’s high lipophilicity and fast permeation through lipid membranes [8,13].

**Figure 1:**
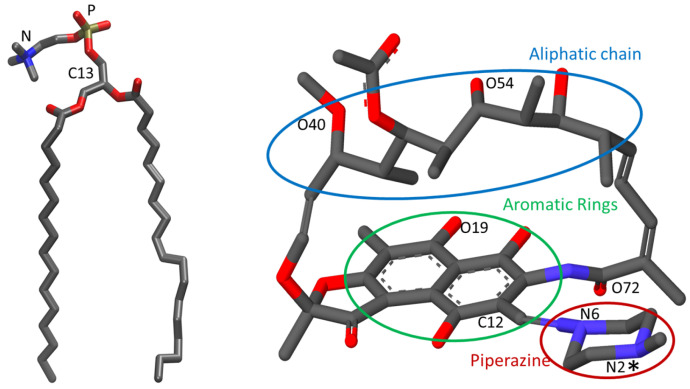
Structure of POPC (**left**) and the *an* species of rifampicin ((**right**); the *zw* species is protonated in the piperazine’s nitrogen atom N2, labeled with an *). Grey, red, blue, and yellow colors indicate carbon, oxygen, nitrogen, and phosphorus atoms, respectively. Numbering for selected atoms (mentioned in the text) is also shown for both molecules.

In this work, we aim to further understand rifampicin’s ability to “escape the rules of five” by studying its interaction with 1-palmitoyl-2-oleoyl-*sn*-glycero-3-phosphocholine (POPC, Figure 1, left) lipid bilayers using a combination of molecular dynamics (MD) simulations and experimental biophysical methodologies. POPC was chosen as a first approach for a simple membrane to mimic biomembranes in general. This is justified by the high abundance of phosphatidylcholines in eukaryotic cell membranes [14,15,16,17,18,19] and the prevalence of the combination of palmitoyl and oleoyl acyl chains in the membrane phospholipids [17,18,19,20]. Mimicking eukaryotic membranes is relevant in this work because of our focus on the interactions of this antibiotic with the host membranes, which determine its efficient absorption and high bioavailability. The membrane affinity of rifampicin is determined by isothermal titration calorimetry (ITC), which also provides insights into changes in rifampicin’s ionization state upon membrane association. MD simulations reveal details on the interactions established with the lipids, location of rifampicin groups within the membrane, and effects on membrane local order. We also assess the impact on membrane barrier properties by characterizing carboxyfluorescein leakage in the presence of increasing concentrations of rifampicin. Finally, we employ umbrella sampling simulations to obtain the free-energy profile of rifampicin’s permeation through the membrane, providing a deeper understanding of its membrane behavior and pharmacokinetic properties.

## 2. Materials and Methods

### 2.1. Materials and Experimental Methods

POPC was acquired from Avanti Polar Lipids, Inc. (Alabaster, AL, USA) and all other reagents and solvents were of the highest commercially available purity from Sigma-Aldrich Química S.A., Sintra, Portugal. Rifampicin 99.95% was from Parafarm (Buenos Aires, Argentina), and carboxyfluorescein (CBF) was from Acros Organics (Geel, Belgium). Large unilamelar vesicles (LUVs) of POPC were prepared following the procedure described previously [21]. In brief, aqueous suspensions of the lipid were prepared in Tris buffer 10 mM pH = 7.4 with 0.15 M sodium chloride, 1 mM EDTA, and 0.02% NaN_3_ (hereafter designed by Triz-buffer), or in phosphate-buffer or HEPES-buffer that contained 10 mM sodium phosphate or 10 mM HEPES, respectively, and all additional compounds and properties as indicated for Triz-buffer. The lipid suspension was subjected to several cycles of vortex/incubation at room temperature and 3 cycles of freeze and thaw (with an extrusion step between each cycle), and further extruded with a minimum of 10 passes, through two stacked Nucleopore polycarbonate filters with a pore diameter of 0.1 μm. A molar volume of 0.756 dm^3^ mol^−1^ was considered for POPC in the LUVs [22].

Titrations were performed on a VP-ITC from MicroCal (Northampton, MA, USA) at 25 °C, injection speed 0.5 μL s^−1^, stirring speed 459 rpm, and reference power 10 μcal s^−1^. The titrations were performed with additions of 10 μL of the LUV suspension into the rifampicin solution in the sample cell. All solutions were previously degassed for 10 min, and the titrations were performed at 25 °C. The obtained thermogram was integrated using the data analysis software Origin 7.0 as modified by Microcal to deal with ITC experiments and the resulting differential titration curve was analyzed with the appropriate equations using Microsoft Excel^®^ and Solver^®^. The concentrations in the cell were calculated taking into account the volume that overflows the cell due to the addition of solution from the syringe, considering that overflow is faster than mixing, as previously described [21,23]. The association of rifampicin with the POPC LUVs was also characterized by UV-vis spectroscopy (Unicam UV530 spectrophotometer, Cambridge, UK), following the red-shift in the absorption spectra that accompanies rifampicin association with the lipid membrane. Rifampicin at 10 μM was prepared in Trizma-buffer and increasing amounts of LUVs in the same buffer were added. The solutions were allowed to equilibrate for 15 min, prior to the measurement of the UV-vis spectrum. The overall charge of rifampicin was also evaluated by measuring the zeta potential (Zetasizer Nano ZS, Malvern, UK) of POPC LUVs (0.1 mM) equilibrated with increasing concentrations of rifampicin in phosphate buffer without added salts. The quantitative relation between the zeta potential measured and the surface charge density was obtained using the Gouy−Chapman theory following the procedure described in Appendix A.

The effect of rifampicin on the POPC membrane barrier properties was evaluated through the effect on carboxyfluorescein (CBF) leakage encapsulated at 5 or 50 mM following two approaches: (i) the usual procedure with efflux measured indirectly from the fluorescence increase that accompanies the dilution of CBF when permeating out of LUVs encapsulating CBF at a high concentration [24,25]; and (ii) directly through the quantification of CBF that remains encapsulated in the LUVs after LUV separation by size exclusion chromatography (Zeba^TM^ spin desalting columns or 96-well filter plate, both 7 k MWCO, from Thermo Fisher Scientific, Waltham, MA, USA).

### 2.2. Molecular Dynamics Simulations

MD simulations were carried out for both anionic (*an*) and zwitterionic (*zw*) ionization states of rifampicin (Figure 1, right).

Fully hydrated (50:1 water/lipid ratio) POPC bilayers were assembled with MemGen [26]. The united-atom GROMOS 54A7 force field was used [27], with lipid parameters taken from Poger and Mark [28]. The water SPC model was used [29]. Topologies for both *an* and *zw* rifampicin were obtained using Automated Topology Builder [30,31], with refinement of atomic charges using GAMESS-US version 2018 R1 [32]. Optimized geometries of *an* and *zw* rifampicin were obtained by density functional theory (DFT) using the hybrid exchange–correlation functional B3LYP [33,34] together with the 6–31G* basis set. Frequency analysis subsequently performed confirmed each optimized geometry as an energy minimum by the absence of imaginary frequencies. Partial charges for optimized rifampicin were calculated from a least-squares fit to the electrostatic potential obtained at the same theory level, according to the Kollman and Singh schemes [35,36]. For *an* simulations, system neutralization was carried out by adding the required number of sodium ions. All simulations were carried out with GROMACS 2019.4 [37,38,39,40]. The same software was employed for analysis, except for the determination of POPC acyl chain order parameters for varying distances to the closest rifampicin molecule, which was carried out using in-house software. For snapshot and trajectory visualization, VMD was used [41].

For each ionization state, two simulation boxes were prepared for the unrestrained simulations, one with two rifampicin molecules located in the center of the bilayer (*c* systems), and another with four rifampicin molecules dispersed in the aqueous medium (*w* systems). The former systems were prepared by pulling rifampicin molecules from the water medium to the center of the bilayer, at a rate of 0.0001 nm/ps and a force constant of 500 kJ mol^−1^ nm^−2^. Both *c* and *w* systems were energy-minimized using the steepest descent algorithm and underwent two 100-ps simulations with 1 fs integration step, in the *NVT* and *NPT* ensembles, respectively. Following these equilibration steps, production runs of 1000 ns (*c* systems) or 2000 ns (*w* systems) were carried out with 2 fs integration steps in the *NPT* ensemble at 1 atm and 298.15 K, controlled with the Parrinello-Rahman barostat [41] and Nosé-Hoover thermostat [42,43], with time constants of 2.0 ps and 0.5 ps, respectively. Semiisotropic pressure coupling was used. Bond lengths were constrained to their equilibrium values, using the SETTLE algorithm [44] for water and the LINCS algorithm [45] for all other bonds. Van der Waals interactions were cut off at 1.0 nm. Coulomb interactions were calculated using the Particle Mesh Ewald method [46], with a cut-off of 1.0 nm for the real space component.

For umbrella sampling (US) simulations, systems with 200 POPC molecules and an 80 water/lipid molecular ratio were prepared and equilibrated as described above. The free energy of the system, as a function of the reaction coordinate (defined as the distance between the center of mass of the rifampicin molecule and the local center of mass of the POPC bilayer, i.e., calculated using only the POPC molecules whose locations in the bilayer plane were contained in a 1.5 nm radius cylinder centered on the solute), is derived from the potential of mean force (PMF), obtained from US simulations [47]. For this purpose, a first solute molecule, initially in the aqueous phase, is pulled to the center of the bilayer (*z* = 0), with a rate of 0.0005 nm/ps and a force constant of 500 kJmol^−1^ nm^−2^. A second solute molecule is then put at a location *z* = −4.0 nm, inside the aqueous phase. Subsequently, a second pulling run was carried out, gently pulling both molecules towards positive *z* values, using the same speed and force constant of the previous step, starting from *z* = 0 and ending at *z* = 4.0 nm for the first molecule (*cw* direction), and starting from *z* = −4.0 and ending at *z* = 0 for the second one (*wc* direction). From this simulation, 41 configurations were extracted in which the first and second molecules were approximately in each of the transverse positions in the [0, 4.0 nm] and [−4.0 nm, 0] intervals, respectively, spaced 0.1 nm apart. This procedure was chosen to obtain the initial configurations for the US runs. For the two rifampicin forms, each of these 41 systems was simulated for 120 ns, using the same conditions as in the unrestrained runs but imposing a harmonic restraint potential, centered in the reference position, with a force constant of 3000 kJmol^−1^ nm^−2^. The resulting simulations were checked for convergence and analyzed using the Weighted Histogram Analysis Method [48,49] to produce the PMF profiles.

## 3. Results and Discussion

### 3.1. Experimental Results for the Interaction of Rifampicin with POPC Bilayers

#### 3.1.1. Association of Rifampicin with POPC LUVs

**Figure 2:**
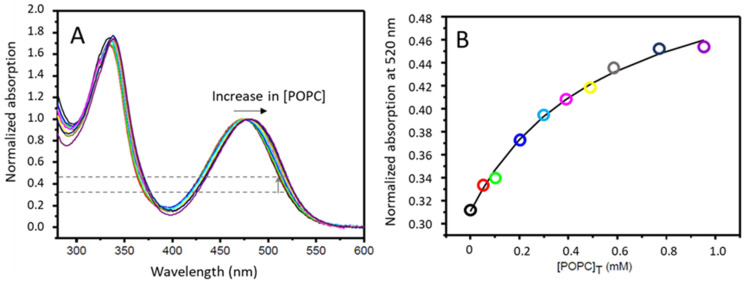
Plot (**A**) UV-vis spectra of rifampicin in the presence of increasing concentrations of POPC LUVs, in saline phosphate buffer at 37 °C. The total concentration of rifampicin is 10 μM and the spectra were normalized at the maximum near 500 nm. The grey dashed lines indicate the minimum and maximum value of the normalized absorption at 520 nm, and the vertical grey arrow indicates the variation with the increase in the concentrations of POPC. Plot (**B**) Normalized absorption at 520 nm (from the spectra shown in plot (**A**)) as a function of the total concentration of POPC. The symbol colors correspond to the spectra shown in plot (**A**) from 0 (black) to 0.95 mM (purple), and the line is the best fit of the equation for a simple partition.

The partition coefficient of rifampicin between Triz-buffer and POPC LUVs has been previously characterized by ITC at 25 °C, showing a moderately high affinity for the membrane ($K_{P}\;$= 2 ± 1 × 10^3^) and a negative enthalpy of interaction (${∆H}_{obs}^{o}$= −7 ± 3 kJ mol^−1^), suggesting stabilizing interactions with the lipid membrane [21]. From the partition coefficient of rifampicin to different organic solvents, it was suggested that association with lipid membranes could stabilize the neutral form of rifampicin, justifying its relatively high lipophilicity and fast permeation [7,8]. In agreement with the behavior observed in non-protic solvents, the UV-vis absorption spectra of rifampicin changed upon association with the POPC membrane, and this was followed to characterize the partition coefficient, Figure 2. The value obtained (3.2 × 10^3^) is in good agreement with that obtained by ITC although somewhat larger, possibly due to the different temperature. The bathochromic shift observed upon association with the lipid membrane is also in good agreement with that obtained in non-polar media [7], supporting the interpretation that the neutral form of rifampicin is stabilized when associated with the POPC membrane.

**Figure 3:**
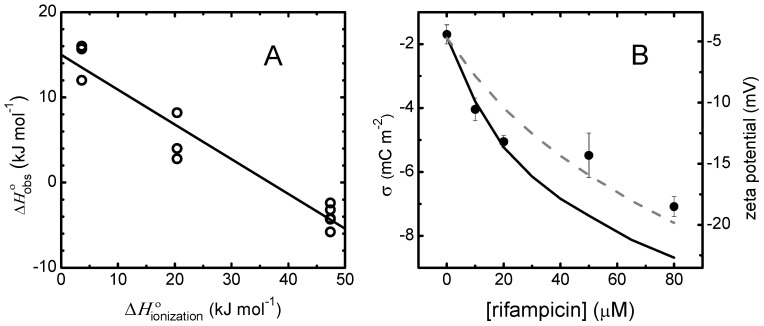
Variation in the ionization state of rifampicin upon association with POPC LUVs. Plot (**A**): Observed calorimetric enthalpy variation obtained by ITC for the association of rifampicin at 20 μM with POPC LUVs in phosphate, HEPES, or Triz buffer, with $∆H_{ionization}^{o}\;$ = 3.6, 20.4, or 47.4 kJ mol^−1^ respectively [51,52], at 25 °C. The line is the best fit of Equation (A1) corresponding to $∆H_{P}^{o}\;$= 15 ± 2 kJ mol^−1^ and $∆nH^{+}\;$= −0.41. Plot (**B**): Variation in the zeta potential (right axis) and corresponding surface charge density (left axis) of POPC LUVs in 10 mM phosphate buffer pH = 7.4 without additional added salts, at different concentrations of rifampicin. The surface charge density was calculated from the partition coefficient and the Gouy−Chapman formalism [23,53,54]. The dashed grey line corresponds to the best fit of the surface charge density obtained at all rifampicin concentrations and the continuous black line corresponds to the best fit at low rifampicin concentrations only, assuming the intrinsic $K_{P}$ obtained by ITC and adjusting rifampicin global charge, leading to $z_{Rif}^{M}\;$= −0.43 and −0.77, respectively (see Appendix A, Appendix A.2, for details).

To provide more direct insights regarding the changes in rifampicin ionization upon association with lipid membranes, we have characterized the thermodynamics of interaction with POPC LUVs in aqueous media with the pH controlled with buffers differing in their ionization enthalpy, namely, phosphate, HEPES, and Triz buffer [50]. The results obtained are represented in Figure 3A, and show a strong dependence of the observed calorimetric enthalpy variation ($∆H_{obs}^{o}$) on the enthalpy of ionization of the buffer ($∆H_{ionization}^{o}$), $∆H_{obs}^{o}\;$= −4 ± 1 kJ mol^−1^ in Triz buffer, 5 ± 3 kJ mol^−1^ in HEPES buffer, and 15 ± 2 kJ mol^−1^ in phosphate buffer. This dependence stems from the exchange of H^+^ with the buffer due to rifampicin association with the membrane, and to the different ionization enthalpies of the buffers (47.4, 20.4, and 3.6 kJ/mol for Trizma, HEPES, and phosphate, respectively) [51,52]. From the dependence of $∆H_{obs}^{o}$ on $∆H_{ionization}^{o}$, one can obtain the intrinsic enthalpy variation for rifampicin partition between the aqueous medium and the POPC membrane ($∆H_{P}^{o}\;$= 15 ± 2 kJ mol^−1^) and the number of H^+^ exchanged between rifampicin and the buffer upon partition to the membrane ($∆nH^{+}\;$= −0.41 ± 0.05), as shown in Equation (A1). The positive intrinsic enthalpy variation obtained shows that association with the membrane is not stabilized by enthalpy, and supports the small increase in membrane affinity observed at 37 °C.

The results presented in Figure 3A show that rifampicin releases H^+^ upon association with the POPC membrane, but by itself, this does not provide the charge of rifampicin. To calculate the overall charge of rifampicin when associated with the membrane, one needs to know its overall charge when in the aqueous medium. Considering the $pK_{a}$ values reported by Ermondi and co-workers ($pK_{a}^{Phenol}=3.0$ and $pK_{a}^{Piperazine}$ = 7.5) [7], at pH = 7.4, the phenol group is fully deprotonated, while the piperazine group is 56% in the protonated state and 44% in the neutral form, leading to an overall average charge for rifampicin in the aqueous medium ($z_{Rif}^{W}$) equal to −0.44. The protons released upon partition to the membrane are, thus, due to stabilization of the neutral form of piperazine, leading to a predicted global average charge for rifampicin associated with the POPC membrane ($z_{Rif}^{M}$) equal to −0.85. When using the $pK_{a}$ values reported by Gallo and co-workers ($pK_{a}^{Phenol}=1.8$ and $pK_{a}^{Piperazine}$ = 7.9) [12], the calculated $z_{Rif}^{W}$ is −0.24, leading to a predicted $z_{Rif}^{M}$ of −0.65. To provide a better estimate of the rifampicin charge, the zeta potential of the LUVs was measured in the presence of increasing concentrations of rifampicin, with the results shown in Figure 3B. In the absence of rifampicin, the zeta potential is slightly negative, −4.4 ± 1 mV (corresponding to a surface charge density σ = −1.8 mC m^−2^), and becomes increasingly more negative in the presence of rifampicin, being −19 ± 1 mV at 80 μM rifampicin (corresponding to σ = −8.7 mC m^−2^). From the best fit of the surface charge density obtained at low rifampicin concentrations, an overall charge of −0.77 ± 0.05 is obtained for rifampicin associated with the POPC membrane, in good agreement with the predictions from $∆nH^{+}$ obtained by ITC and the ${pK}_{a}$ values reported by Gallo. The formalism used is explained in detail in Appendix A. Briefly, the surface charge density is calculated from the partition coefficient for a tentative rifampicin overall charge, which allows the calculation of the surface potential. The surface potential is also calculated from the observed zeta potential, and the overall charge is adjusted until convergence [9,21,23,55,56,57,58,59,60,61]. The surface charge observed at high rifampicin concentrations is much lower than predicted, with the overall best fit (dashed grey line) underestimating the surface charge density at low rifampicin concentrations. This indicates that the overall charge of rifampicin associated with the membrane becomes less negative, or that the affinity of rifampicin for the membrane decreases more than predicted from the repulsive electrostatic effects, possibly due to membrane perturbation and/or saturation with rifampicin [62].

From studies of rifampicin ionization behavior and partition between aqueous media and different non-polar solvents, it has been suggested that in non-protic solvents, the neutral form is stabilized relative to the zwitterion, and that the cationic form may be relevant in non-polar environments [7]. In contrast, the results presented in Figure 3 show that it is the negative species that is stabilized when rifampicin associates with the POPC membrane. This highlights the distinctive properties of lipid membranes and the limitations of using homogeneous solvents as biomembrane models. The negative charge of rifampicin when associated with lipid membranes contributes to the strong decrease in affinity observed for membranes containing negatively charged lipids [21]. Although this observation is unexpected given rifampicin’s antibiotic properties and the high abundance of negatively charged lipids in bacterial membranes [63,64,65,66], it reflects the non-membrane-related mechanism of rifampicin antibiotic activity [67]. According to the current model for membrane permeation, the partition/diffusion model [68], the lower affinity of rifampicin for the negatively charged membranes is expected to proportionally decrease the rate of permeation. However, given the high permeability coefficient through neutral POPC membranes observed for rifampicin ($logP_{app}$ = −4.27 [8]), a moderate permeability would still be expected in the case of negatively charged membranes such as those of the target bacterial cells. Moreover, the extensive membrane perturbation by rifampicin (Section 3.1.2 and Section 3.2.3) points to the possibility of an alternative mechanism of permeation, involving the formation of transient membrane defects rather than diffusion through the membrane non-polar core [8,24,68,69,70,71,72,73,74,75,76,77]. Efficient rifampicin permeation through negatively charged membranes is, therefore, expected, in agreement with its high antibiotic efficiency.

**Figure A1:**
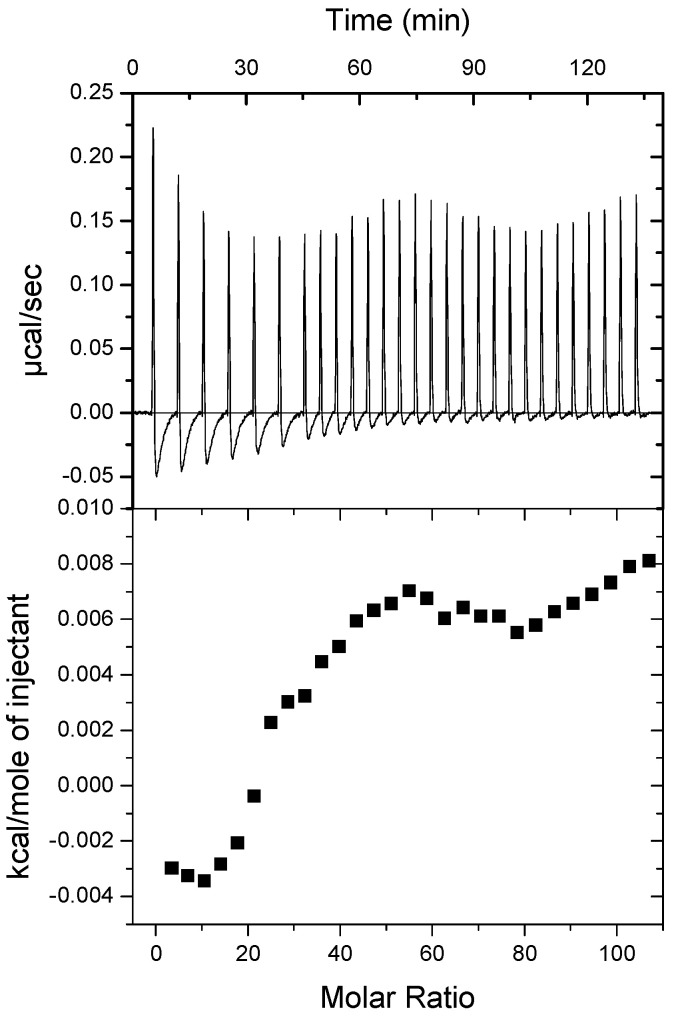
Thermogram obtained for the titration of 100 μM rifampicin with POPC LUVs in Triz buffer at 25 °C. The raw thermogram is shown in the upper plot, and the heat per injection is shown in the lower plot as a function of the molar ratio in the cell (total rifampicin/total lipid).

Insight regarding membrane perturbation by rifampicin may also be obtained from the analysis of the thermodynamics parameters for membrane association. The variation in the Gibbs free energy upon partition may be calculated from the intrinsic partition coefficient ($∆G_{P}^{{}^{\circ}}\;$= −19 kJ mol^−1^), and together with the enthalpy variation ($∆H_{P}^{{}^{\circ}}\;$= 15 kJ mol^−1^), allows calculation of the entropy variation of the system ($T∆S_{P}^{{}^{\circ}}\;$= 34 kJ mol^−1^). The association of rifampicin with the POPC bilayers is, thus, stabilized by a large increase in the entropy of the system, not by the establishment of favorable interactions between rifampicin and the membrane lipids. The relatively high solubility of rifampicin in aqueous media suggests that this behavior is not due to a strong contribution of the hydrophobic effect. Instead, the increase in entropy reflects perturbation of the membrane by rifampicin. The association of rifampicin has been characterized at different total concentrations and a strong decrease is observed in the partition coefficient that cannot be justified only by electrostatic effects. The decrease in membrane affinity is accompanied by a small variation in the enthalpy variation that becomes more favorable. This suggests the establishment of additional interactions and a complex behavior at high rifampicin concentrations. In fact, at 100 μM rifampicin, the thermogram cannot be described by a simple partition, and the heat evolved after POPC addition to rifampicin shows endothermic and exothermic processes (Figure A1), suggesting extensive effects on the membrane properties. This is further explored in the next sub-section, with the effect of rifampicin on the rate of CBF leakage.

#### 3.1.2. Perturbation of the Membrane Barrier Properties by Rifampicin

**Figure 4:**
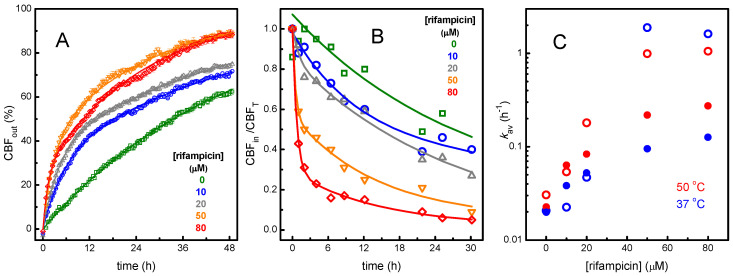
Plot (**A**)—CBF leakage calculated from the increase in CBF fluorescence, Equation (A10), following 100 μL of LUVs solution at a lipid concentration of 0.03 mM with CBF encapsulated at 50 mM and incubated at 50 °C. Plot (**B**)—Time dependence of the fluorescence intensity of the LUVs with encapsulated CBF, after incubation of the LUVs suspension at 50 °C and separation of the LUVs by size exclusion chromatography. The initial concentration of CBF inside the LUVs is 5 mM and the lipid concentration is 0.2 mM. The lines in plots A and B are the best fit of a bi-exponential function. Plot (**C**)—Dependence of the average rate constant for CBF leakage with the concentration of rifampicin, for leakage of CBF encapsulated at 50 mM followed indirectly through the increase in fluorescence when incubated at 50 °C (●) and at 37 °C (●), or directly after separation of the LUVs fraction containing CBF encapsulated at 5 mM when incubated at 50 °C (**◯**) and at 37 °C (**◯**). Note the logarithmic scale in the ordinate axis.

**Figure A2:**
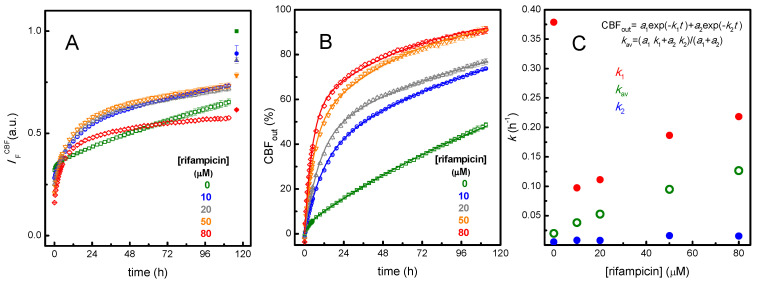
Plot (**A**) Variation in the fluorescence intensity of CBF ($\lambda_{exc}=420\;nm,\;\lambda_{em}=520\;nm$) initially encapsulated in POPC LUVs at 50 mM in PBS, in the presence of increasing concentrations of rifampicin, for a lipid concentration of 0.06 mM and incubation at 37 °C. The sharp increase in fluorescence at 110 h is due to the addition of Triton X-100 at a final concentration of 1%. The data correspond to the average of 3 replicates; the standard deviation is usually smaller than the symbol size and is also shown. Plot (**B**) Corresponding % of CBF outside the LUVs calculated from Equation (A10). The lines are the best fit of a bi-exponential function. Plot (**C**)—Dependence of the rate constants for CBF leakage with the concentration of rifampicin.

**Figure A3:**
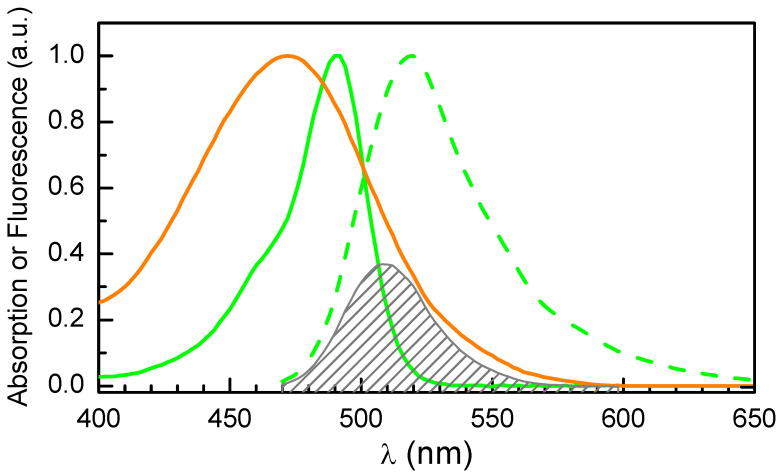
Absorption and fluorescence spectra of CBF and rifampicin. The absorption spectra are shown as continuous lines (**―** for CBF, and **―** for rifampicin), and the fluorescence spectra of CBF is shown by dashed lines (**− −**). The shape of the overlap integral between CBF emission and rifampicin absorption is shown in grey.

CBF encapsulated at 50 mM inside POPC LUVs undergoes efficient self-quenching and its fluorescence intensity increases strongly as it leaches from the LUVs into the outer aqueous medium [78]. The fluorescence corresponding to full equilibration was obtained after breakdown of the LUVs by the addition of triton X-100 to a final concentration of 1%. The results obtained at 37 °C, in the absence and presence of different concentrations of rifampicin, are shown in Figure A2 and show that rifampicin increases the rate of CBF leakage. As explained in detail in the appendix, although a clear trend is observed, a closer inspection of the raw data points towards several possible problems with this methodology that may compromise a quantitative interpretation of the results, namely, a strong quenching of CBF fluorescence by rifampicin due to trivial inner filter effects and/or effects dependent on the proximity of rifampicin and CBF such as FRET due to the strong overlap between CBF fluorescence and rifampicin absorption spectra (Figure A3). In an attempt to overcome artifacts due to inner filter effects, FRET, or other effects that lead to nonlinear relations between the fluorescence intensity and the extent of CBF leakage such as a concentration dependent permeability coefficient [79], several additional leakage experiments were performed. Contributions from trivial inner filter effects were minimized by decreasing the light path, and the extent of CBF leakage was evaluated directly through separation of the LUVs from the outer aqueous media by size exclusion chromatography. The latter protocol does not rely on CBF self-quenching when encapsulated, and thus, a lower concentration of encapsulated CBF was used to minimize contributions from a concentration dependent permeability coefficient. To facilitate the experimental execution of the assays, these experiments were performed at 50 °C. The results obtained are shown in Figure 4.

**Figure A4:**
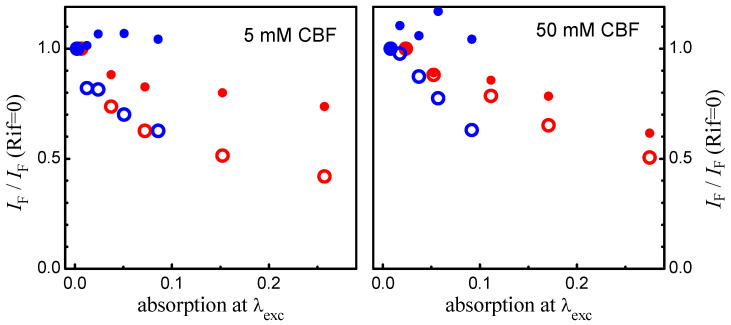
Quenching of CBF fluorescence by rifampicin, for CBF encapsulated in the LUVs at 5 and 50 mM. The hollow symbols (**◯**, **◯**) correspond to the fluorescence intensity immediately after addition of rifampicin to LUVs containing CBF encapsulated, and the filled symbols correspond to the fluorescence intensity after disruption of the LUVs by addition of Triton X-100 (**●**, **●**). The data in red (**◯**, ●) were obtained with 300 μL solution per well, while for data in blue (**◯**, ●) only 100 μL solution per well was used, thus decreasing the light pathlength to 1/3 for.

In the absence of rifampicin, all methods at both CBF concentrations lead to similar rates of CBF leakage, $k_{av}\;$= 0.023 ± 0.005, corresponding to a permeability coefficient of (1.1 ± 0.2) × 10^−11^ cm s^−1^. At low rifampicin concentrations, a small increase in the rate of permeation was observed when the temperature was increased from 37 to 50 °C. However, in the presence of high rifampicin concentrations, this effect was small and not systematic. A small temperature dependence was previously observed for CBF permeation and was interpreted as permeation by transient pores in the lipid bilayer [70]. The addition of rifampicin leads to an increase in the rate of CBF leakage in all conditions, but the rate of permeation observed varies widely, with a larger effect when CBF leakage is followed directly after separation of the LUVs (Figure 4B, and open symbols in plot C). Decreasing the total absorption of the solution does not eliminate the strong quenching of CBF fluorescence by rifampicin when CBF is encapsulated in intact LUVs, while no significant quenching is observed after LUV disruption by the addition of Triton X-100 (Figure A4). This supports the interpretation of a significant fraction of CBF being close to the membrane surface where it may be quenched through FRET to rifampicin. Altogether, the results show that the kinetics of fluorescence increase due to CBF leakage is a simple method that provides qualitative information regarding the effect of rifampicin on the membrane barrier properties but should not be used for a quantitative interpretation due to many possible artifacts. This may be overcome through the separation of the LUV fraction by exclusion chromatography followed by a more direct evaluation of the amount of CBF encapsulated, although having the drawback of being significantly more laborious and expensive.

**Figure A5:**
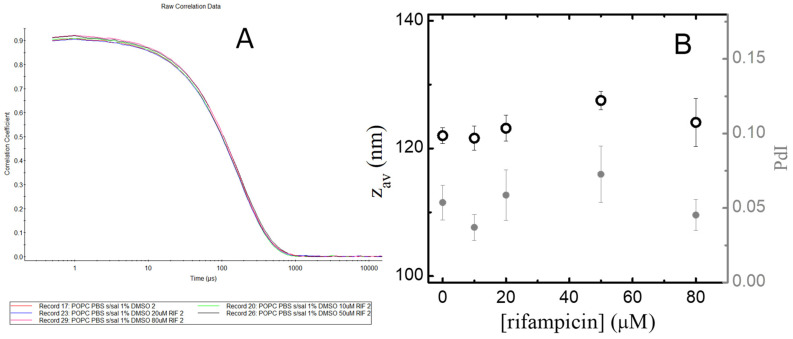
Effect of rifampicin in the size of LUVs. Plot (**A**): autocorrelation of 0.1 mM POPC LUVs in the absence of rifampicin and after incubation with 10, 20, 50, and 80 μM rifampicin. Plot (**B**): average and standard deviation of 3 measurements of the average diameter (◯ and polydispersity index (●) of the LUVs at different rifampicin concentrations.

An interesting observation from the effect of rifampicin in CBF permeation is the bi-exponential behavior observed for rifampicin concentrations larger than 20 μM, with a significant fraction of CBF escaping the LUVs during the first hour of incubation (Figure 4B). A possible interpretation of this effect is the transient strong perturbation of the membrane properties by rifampicin initially only in the outer leaflet of the LUVs at very high local concentrations [80]. From the partition coefficient obtained in Section 3.1, a local concentration of rifampicin higher than 5 mol% is predicted for a total concentration of 50 μM. This high local concentration imposes a significant stress in the membrane and may increase the probability of pore formation and/or pore stability, facilitating the equilibration of rifampicin with the inner leaflet and the release of encapsulated CBF. In fact, when the partition of rifampicin to the LUVs is followed by ITC at these high concentrations, large deviations from the behavior observed in dilute rifampicin solutions are observed. At 50 μM, the variation of the heat profile is still well described by a simple partition, although with a lower affinity and a more negative interaction enthalpy. Strong deviations from the profile expected for a simple partition are, however, observed at 100 μM rifampicin, indicating the presence of additional processes (Figure A1). It should be noted that membrane perturbation by rifampicin does not lead to LUV disruption. This is suggested by the non-instantaneous CBF leakage and was verified through the maintenance of the LUVs size and polydispersity (Figure A5). Further details on the interaction of rifampicin with lipid membranes were obtained by molecular dynamics simulations and are presented in the next section.

### 3.2. Molecular Dynamics Simulation of the Interaction of Rifampicin with POPC Bilayers

As described in Section 2.2, two ionization states of rifampicin (*an*, anion; *zw*, zwitterion) were parameterized and simulated. This choice is justified by the larger prevalence of these species at physiological pH. In membranes, while *an* is the most abundant form, a significant fraction of *zw* is also expected (Figure 3B, Section 3.1.1).

#### 3.2.1. Location and Orientation

**Figure A6:**
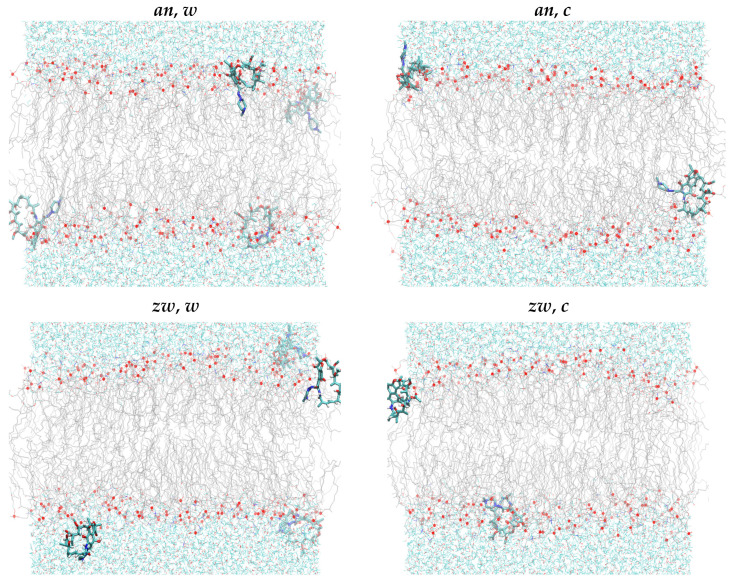
Final configurations of the unrestrained MD simulations. Rifampicin molecules are represented in thicker lines. Water molecules, POPC acyl chains, and POPC P atoms are shown as cyan lines, grey lines, and thick red dots, respectively.

Figure A6 in Appendix B illustrates the final configurations of the four unrestrained simulations (after 2000 ns for the *wc* simulations, and after 1000 ns for the *cw* simulations). These snapshots show all rifampicin molecules in interaction with the POPC bilayer, located mostly near the headgroup region. Occasionally, more external (e.g., molecules in the lower leaflet in the *zw*, *w* snapshot) or internal (e.g., molecule in the lower leaflet in the *an*, *c* snapshot) locations are apparent. In both cases, it is clearly visible that rifampicin molecules drag with them lipid headgroups to regions outside or inside (respectively) those where they would normally reside. These observations, taken from single configurations, already point to the establishment of strong lipid–rifampicin interactions and the possibility of significant rifampicin-induced membrane perturbation. These points are addressed in detail in Section 3.2.2 and Section 3.2.3, respectively.

**Figure A7:**
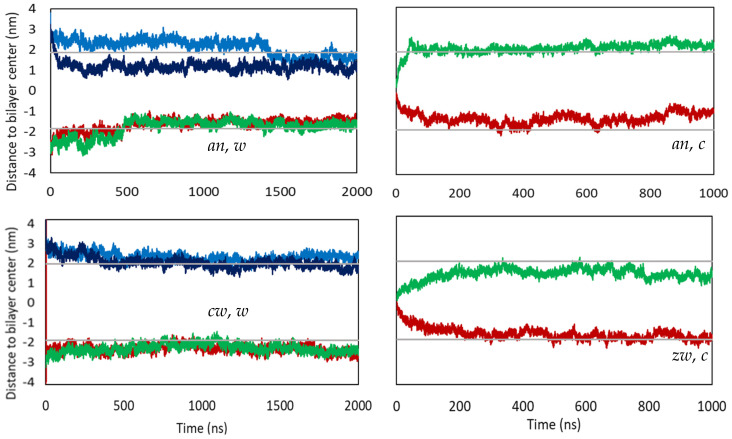
Time variation of the transverse distance to the center of the bilayer of the center of mass of individual rifampicin molecules (each depicted in a different color). Horizontal grey lines (near ±2 nm) indicate average locations of the POPC P atoms in the corresponding leaflet.

Rifampicin location can be quantitatively characterized by the transverse location of the center of mass, relative to that of the POPC bilayer, as shown in Figure A7. In the initial configuration, solute molecules were placed in the water medium, outside the bilayer headgroup region in the *w* simulations, or near the center of the bilayer in the *c* runs. During the simulations, solutes move in the *z* direction (normal to the bilayer plane), to adopt a final location near the interface region. From these results, it is already clear that different molecules, even in the same simulation, display distinct behaviors, with some molecules ending up with their center of mass clearly inside the location of the POPC phosphorous atoms, while others stay in a more external position. With a single exception (one solute in the *an*, *w* simulation, Figure A7), no significant relocations are apparent after 500 ns in all simulations. For this reason (unless specified otherwise), analysis of the unrestrained MD simulations concerned the last 1500 ns or 500 ns of each *w* or *c* run, respectively.

**Figure A8:**
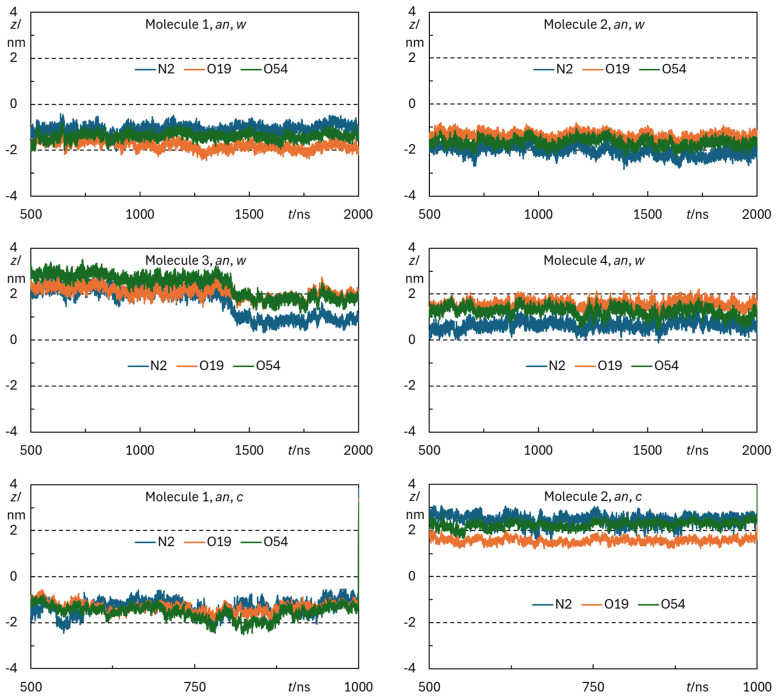
Time variation of the transverse distance to the center of the bilayer of selected rifampicin *an* atoms. Each panel concerns an individual rifampicin molecule of a given identified simulation.

**Figure A9:**
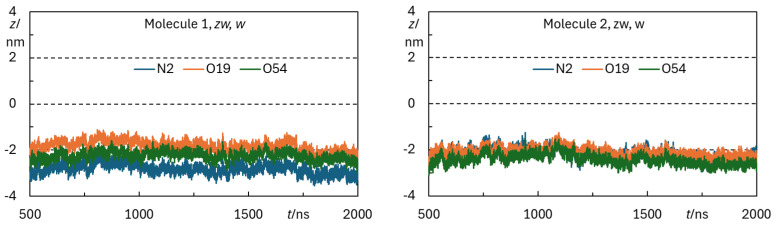

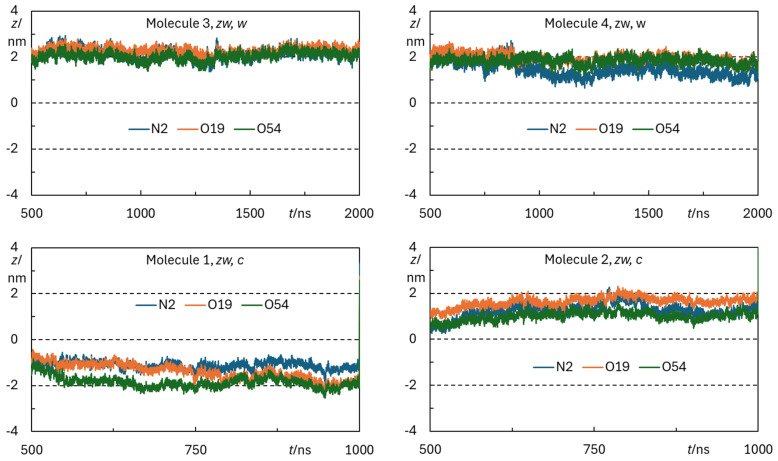
Time variation of the transverse distance to the center of the bilayer of selected rifampicin *zw* atoms. Each panel concerns an individual rifampicin molecule of a given identified simulation.

Figure A8 and Figure A9 illustrate the variation of the transverse position of selected rifampicin atoms in the *an* and *zw* simulations, respectively, during the analysis time range. These plots show that, for most molecules, the orientation in the membrane does not change during the simulation, as atoms located in distinct regions of rifampicin keep their relative positions almost unaltered. It appears that, upon interacting with the bilayer, the molecules establish interactions that are maintained throughout the simulation, locking them in the same basic conformation. Therefore, in the time scale of these simulations, rifampicin rotation is highly impeded. An exception to this behavior is observed for molecule 3 of the *an*, *w* simulation (Figure A8). For this molecule, while the position of the phenolic O19 atom is kept essentially unaltered, both the aliphatic chain and (especially) the piperazine group undergo insertion around ~1300 ns.

**Figure A10:**
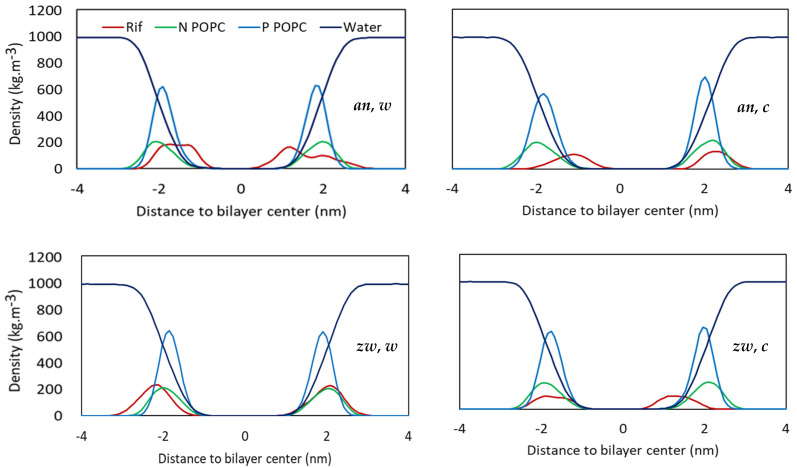
Mass density plots along the direction normal to the bilayer plane of rifampicin (red), water (dark blue), and lipid P and N atoms (light blue and green, respectively).

The preferred interfacial location of rifampicin is also evident in the mass density profiles along the *z* direction, shown in Figure A10. Although the rifampicin peaks are rather broad (reflecting not only heterogeneity in the location of different individual molecules, but also the sheer size of the rifampicin molecule), they are mostly centered around values not far from the position of POPC phosphate (P POPC) and, although some come close, they do not reach the geometric center of the bilayer (*z* = 0). Still, there is considerable diversity, and even within the same simulation, the molecules inserted in the two opposite leaflets may display quite different distributions.

Figure A10 shows that, despite this diversity, there is an acceptable degree of similarity in the distributions of the rifampicin molecules inserted into the two bilayer leaflets in the *w* simulations of both ionization states (Figure A10, left panels). Conversely, and possibly because of the lower number of simulated molecules, the rifampicin distributions in the *c* simulations (Figure A10, right panels) are not symmetrical. For example, in the *an*, *c* simulation, the molecule in the lower leaflet (*z* < 0) adopts clearly internal positions, whereas that on the upper leaflet (*z* > 0) is preferentially located outside the location of the POPC P atoms. For this reason, in the following and unless stated otherwise, we will focus on the *w* simulations of both rifampicin forms.

**Figure 5:**
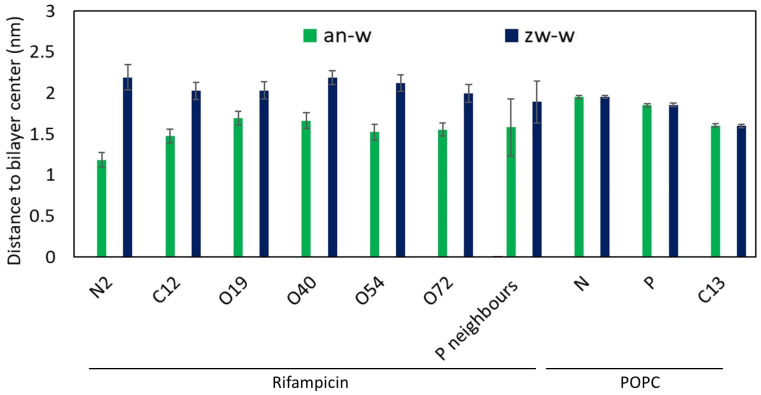
Distance to the center of the bilayer of various reference atoms of rifampicin and POPC (see Figure 1 for definition), and of phosphorous atoms closest to rifampicin (P neighbors, at <0.5 nm). Error bars reflect standard deviations over all analyzed frames of the instant averages among the four rifampicin or 200 POPC molecules.

Looking at the average location of specific rifampicin atoms in different locations along the molecular structure (numbering defined in Figure 1), as shown in Figure 5, some differences between the two ionization states become clear. First, all atoms of the *zw* form appear to have a more external average location than that of their counterparts of the *an* state. Second, all studied atoms of the *zw* form are located, on average, at similar distances to the bilayer center, indicating that, when in this ionization state, the orientation of rifampicin is mostly parallel to the bilayer plane. This is not the case for the *an* form, for which the piperazine ring (represented by N6 in the figure) is the most deeply located part of the molecule. At variance with the zwitterion, this ring is unprotonated and, therefore, electrically neutral in the *an* state, favoring its insertion into the upper lipid acyl chain region of the bilayer. In turn, the insertion of the piperazine moiety in the *an* form effectively pulls the entire molecule to a more internal average, compared to the zwitterion. Overall POPC atom positions (shown in the three sets of columns furthest to the right) do not vary across all studied systems (located around *z* = (1.84 ± 0.03) nm) and are not significantly different from that in the absence of rifampicin (*z* = (1.84 ± 0.01) nm). However, the *z* position of the POPC P atoms closest (at <0.5 nm distance) to rifampicin is considerably reduced (despite the large uncertainty, which stems from the diverse behaviors of individual rifampicin molecules, and the low number of nearest neighboring lipids), particularly in the *an* state. This points to the above commented “dragging” effect visible in Figure A6.

**Figure 6:**
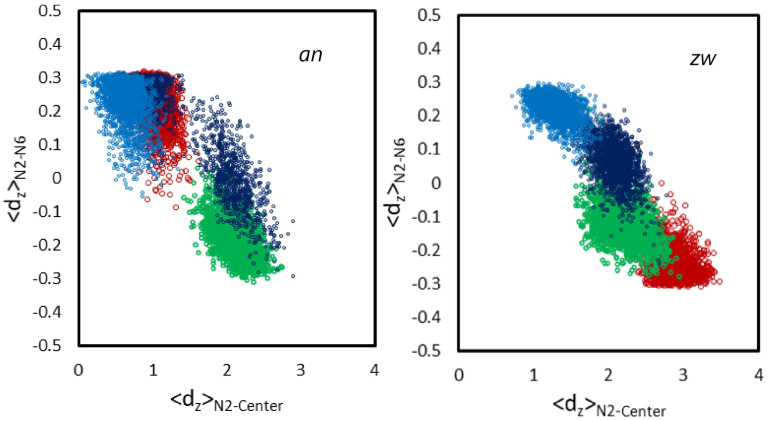
Transverse distance between the N2 and N6 rifampicin atoms, as a function of the transverse distance between the rifampicin N2 atom and the center of the bilayer. Each color concerns a different rifampicin molecule. The whole *w* trajectories were taken into consideration in these plots.

**Figure A11:**
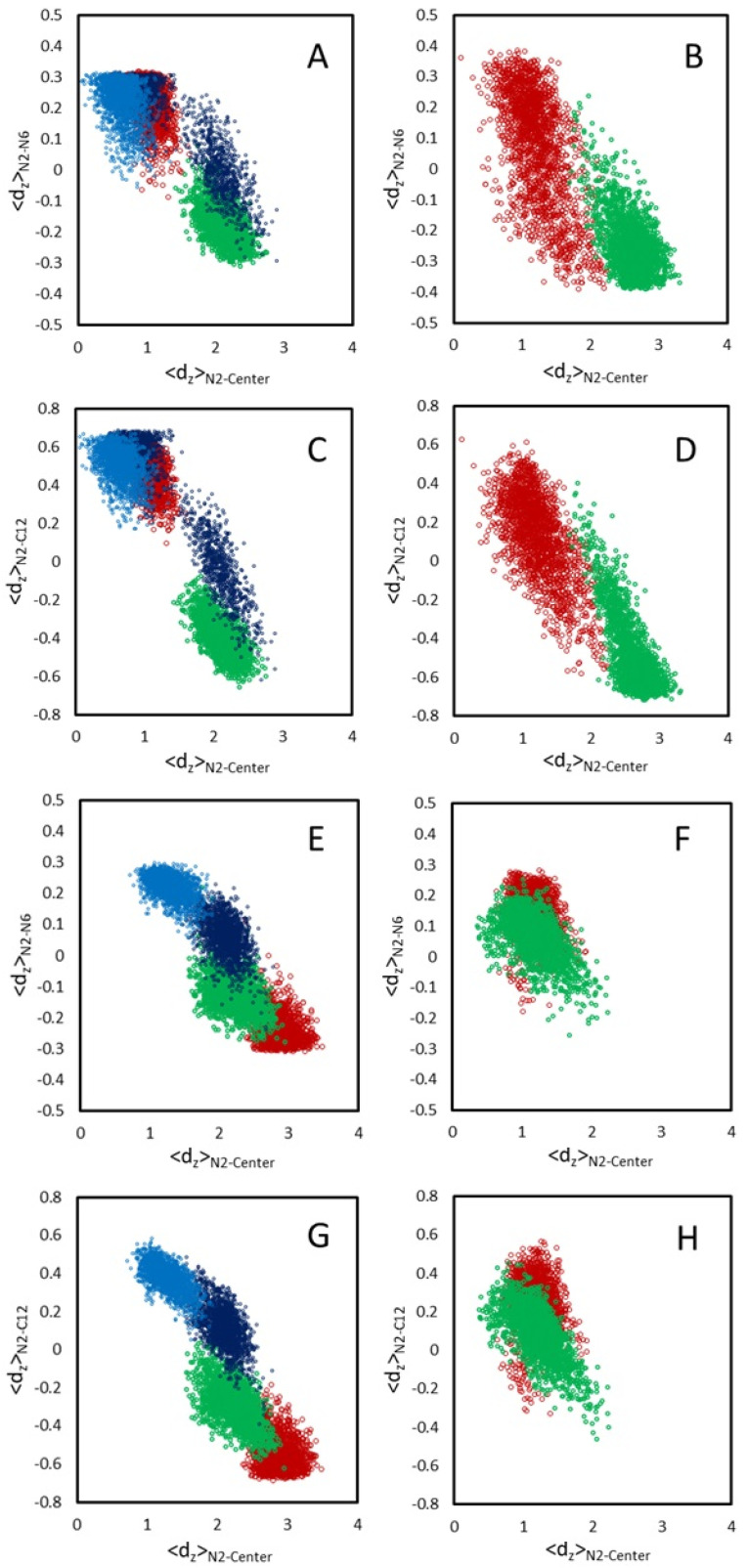
Transverse distance between the N2 and N6 (**A**,**B**,**E**,**F**) or N2 and C12 (**C**,**D**,**G**,**H**) rifampicin atoms, as a function of the transverse distance between the rifampicin N2 atom and the center of the bilayer. The top (**A**–**D**) and bottom (**E**–**H**) panels refer to the *an* and *zw* species, respectively. The left (**A**,**C**,**E**,**G**) and right (**B**,**D**,**F**,**H**) panels refer to the *w* and *c* simulations, respectively. Each color concerns a different rifampicin molecule. The whole trajectories were taken into consideration in these plots.

In a complementary analysis, the correlation between the orientation and the transverse location of the piperazine ring and its linkage to the rest of the molecule was investigated. For this purpose, the two opposing nitrogen atoms of the piperazine ring, N2 and N6, and the aromatic ring atom to which the piperazine moiety is attached, C12, were considered (see structure in Figure 1). The plots in Figure 6 explore the dependency of the transverse distances between N2 and N6 on the transverse distance of N2 to the bilayer center. Each panel in Figure 6 concerns a single *w* simulation, and each set of distinctly colored points concerns a different molecule within that simulation. Figure A11 also shows the dependency of the transverse distances between N2 and the aromatic ring atom to which the piperazine moiety is attached, C12, on the transverse distance of N2 to the bilayer center, including also the *c* simulations. While it is clear that in most simulations, the various molecules have non-equivalent behavior, a correlation pattern is clear. Conformations in which the piperazine group is more internally located (abscissa closer to 0) have higher ordinate values, indicative of an orientation of this ring more aligned with the membrane normal and the lipid acyl chains. In these conformations, the positive ordinates indicate that the N2 end of the piperazine ring has a more internal location compared to N6 (or the aromatic ring atom C12). On the other hand, when the piperazine ring has a more external position (higher abscissae), the ordinates decrease and eventually become predominantly negative, indicating that when the piperazine ring is not inserted in the bilayer, the N2 atom is more external than the opposite N6 atom, or the aromatic ring atom C12. This tendency is essentially conserved across all simulations.

#### 3.2.2. Interactions Between Rifampicin and Lipid Groups

**Figure 7:**
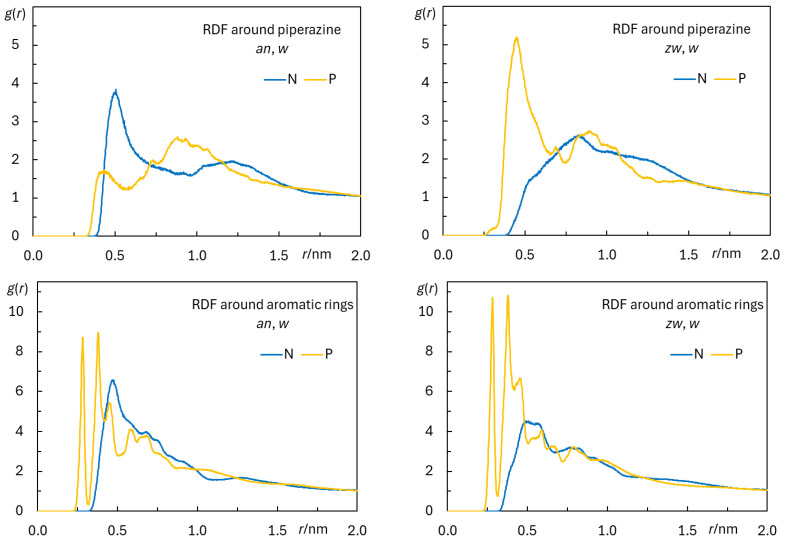
Radial distribution functions *g*(*r*)(RDFs) of POPC P or N atoms around the rifampicin piperazine ring (**top** plots) or aromatic rings (**bottom** plots), in the *an*, *w* (**left** plots) and *zw*, *w* simulations (**right** plots). The final 500 ns of each simulation were taken into consideration in these plots.

**Figure A12:**
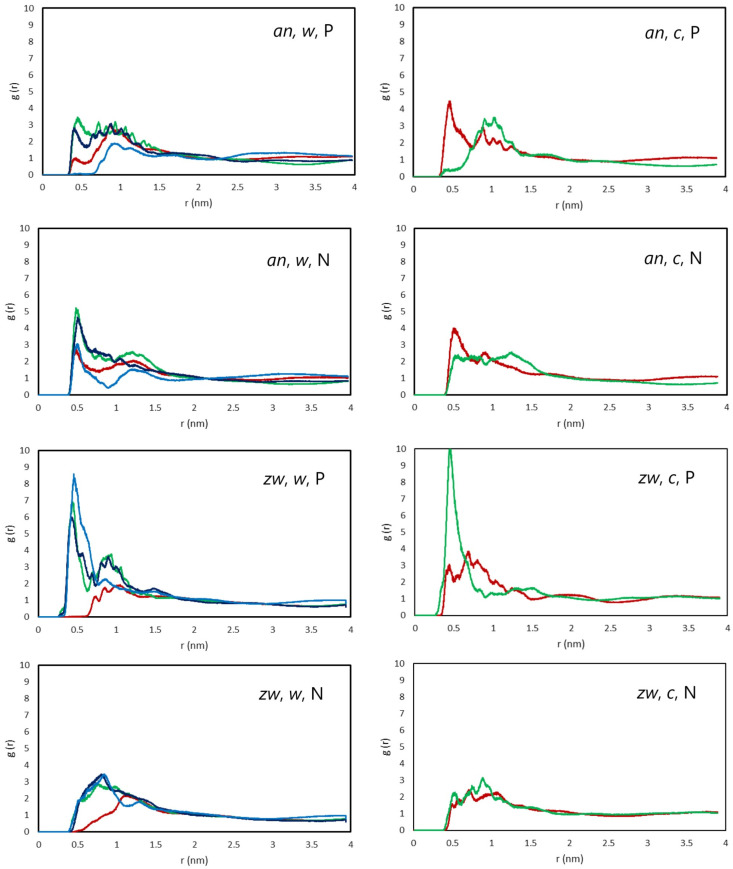
Radial distribution functions (RDFs) of POPC P (first and third rows) or N (second and fourth rows) atoms around the rifampicin piperazine ring. The panels in the top two rows and bottom two rows refer to the *an* and *zw* species, respectively. The left and right panels refer to the *w* and *c* simulations, respectively. Each color concerns a different rifampicin molecule, with an identical color scheme to that of Figure A11. The final 500 ns of each simulation were taken into consideration in these plots.

**Figure A13:**
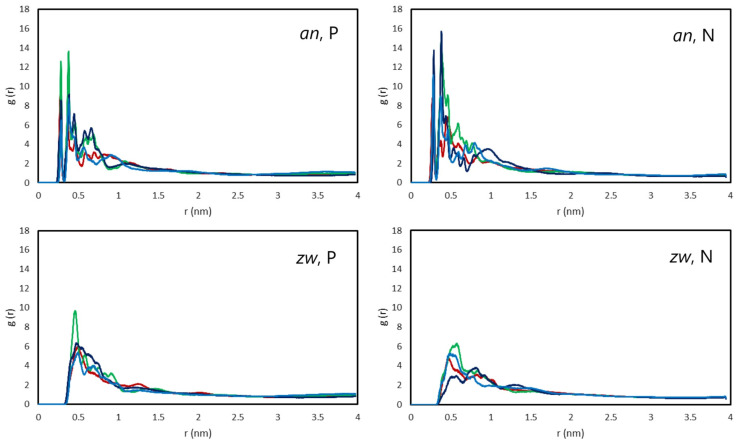
Radial distribution functions (RDFs) of the rifampicin aromatic ring system around the POPC P (**left**) or N (**right**) atoms, calculated for the *w* simulations. The top and bottom panels refer to the *an* and *zw* species, respectively. Each color concerns a different individual rifampicin molecule, with an identical color scheme to that of Figure A11. The final 500 ns of each simulation was taken into consideration in these plots.

Radial distribution functions (RDFs) measure the relative probability *g*(*r*) of finding a particle/group of particles for varying distance *r* to a reference particle/group of particles. They are particularly useful for the characterization of specific intermolecular and intramolecular interactions between atoms and/or atomic groups. Figure 7 depicts the RDFs for the phosphorus and nitrogen atoms of POPC, around the piperazine groups and aromatic rings of rifampicin. RDFs around these groups, also including the *c* simulations, are shown in Figure A12 and Figure A13 for each individual rifampicin molecule.

From the RDFs, it is possible to develop several conclusions. Comparing the two rifampicin states, it is observed that the interaction with POPC P is stronger in the case of piperazine of the *zw* species. In relative terms, the RDF peak for POPC P around piperazine triples its value upon protonation of this group (Figure 7, top panels). This result was expected, because in the *zw* species, the protonated piperazine N6 atom confers a positive charge to this group, enabling favorable electrostatic interaction with the negatively charged phosphate group. Furthermore, due to the protonation of the piperazine nitrogen, it could, in principle, establish hydrogen bonds with the phosphate oxygens (see below). In turn, this protonation of the piperazine in the *zw* species renders its interaction with the positively charged lipid choline group unfavorable, and this is visible in the severe reduction in the peak at ~0.5 nm in the corresponding RDF. Conversely, for the *an* form, the interaction of piperazine with the choline moiety is actually stronger than that with the phosphate.

On the other hand, RDFs of POPC N and P atoms are clearly better defined and have higher values around the aromatic ring system of both forms (Figure 7, bottom panels) in comparison with those around the piperazine groups (note the different ordinate scales in the top and bottom panels of Figure 7). Comparing between the two states, RDFs around the aromatic groups are slightly lower for POPC P atoms and higher for POPC N atoms in the *an* species, compared to the *zw* species, similarly to those around the piperazine group.

**Figure A14:**
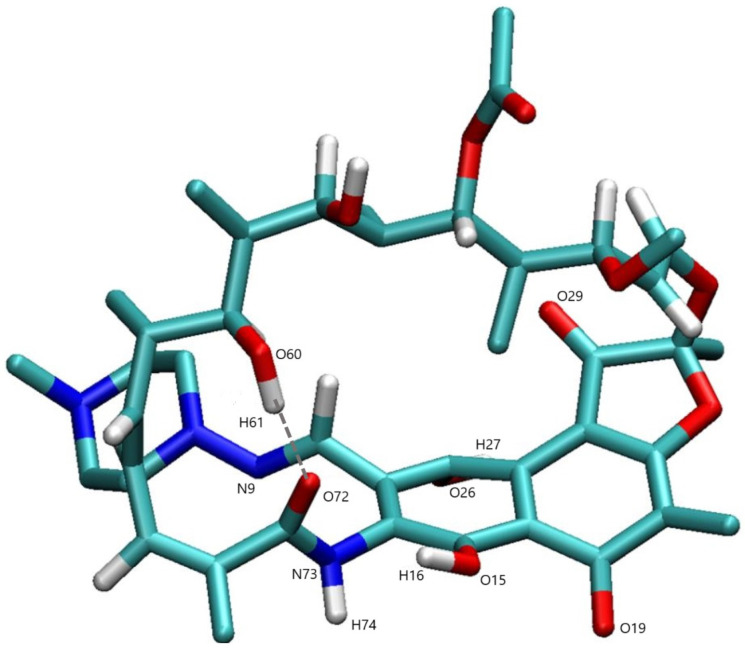

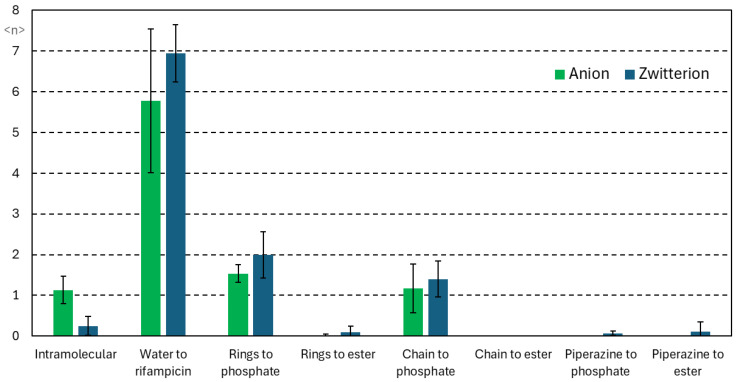
**Top**: structure of rifampicin with selected numbered atoms that could be involved in hydrogen bonding interactions, and in a conformation compatible with the formation of intramolecular H bonds (dotted line indicates one such possibility). **Bottom**: average number of instant H bonds in the *w* simulations involving rifampicin atoms.

Rifampicin possesses numerous oxygen and nitrogen atoms, which can act as hydrogen bond acceptors. Some of these atoms are covalently linked to hydrogen atoms, enabling them to also potentially act as hydrogen bonding donors. Figure A14, top panel, identifies the rifampicin atoms that are capable of establishing this type of interaction and are mentioned in the text.

**Figure A15:**
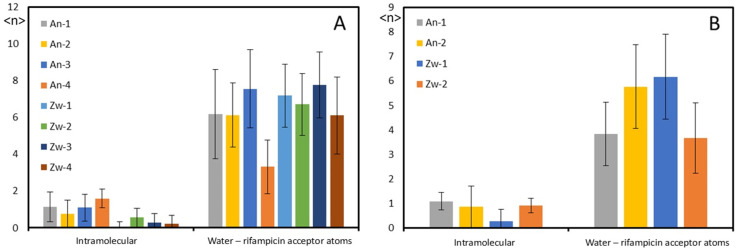
Average number of instant H bonds to rifampicin acceptor atoms. (**A**,**B**) panels refer to *w* and *c* simulations, respectively. Each column refers to an individual rifampicin molecule.

**Figure A16:**
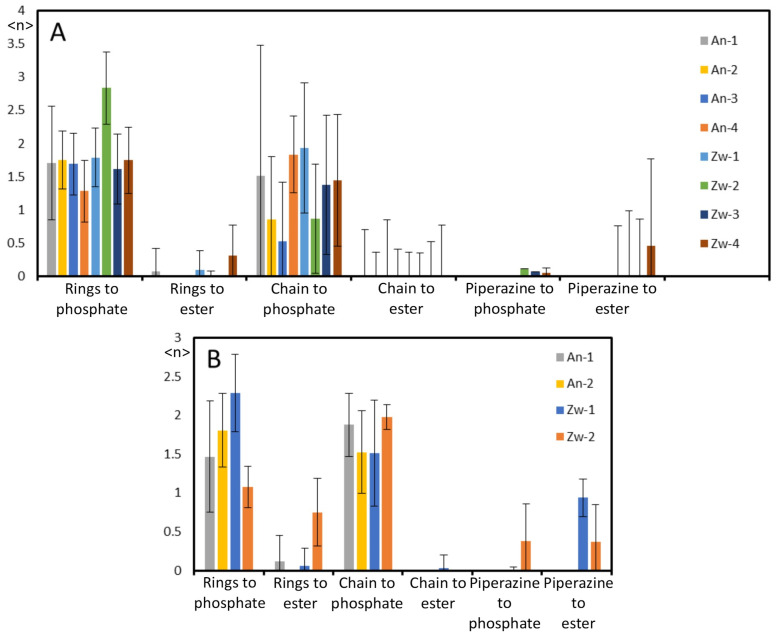
Average number of instant H bonds from rifampicin donor groups to POPC oxygen atoms. (**A**,**B**) panels refer to *w* and *c* simulations, respectively. Each column refers to an individual rifampicin molecule.

The bottom panel of Figure A14 shows average instant number of H bonds per rifampicin molecule, involving both donor and acceptor groups within the same molecule (intramolecular), or where rifampicin acts as an acceptor from water, or where rifampicin acts as a donor to lipid oxygen atoms. More detailed information, including averages for each individual rifampicin molecule, is provided in Figure A15 and Figure A16. On average, rifampicin molecules form four to eight instant H bonds with donor water OH groups. This variability stems from the different transverse locations of each particular molecule, and, on close inspection, those that, on average, reside most internally are the ones that establish the fewest H bonds. Probably because the *an* form tends to have a slightly deeper location in the membrane, the average instant number of H bonds with water is lower than for the zwitterion, although not significantly so.

Given the abundance of H-bond donors and acceptors in its structure, it is not altogether surprising that intramolecular H bonds are common, averaging 1.1 and 0.25 per *an* or *zw* (respectively) at a given instant. Some of the observed interactions lead to the formation of a 6- (N73-H74-N9, O15-H16-O19) or 7- atom ring (O26-H27-O29, O15-H16-O72) involving donor and acceptor atoms that are fairly close in the structure of the molecule. Other observed intramolecular bonds involve donor and acceptor groups that would normally be expected to be more distant from each other, such as O15-H16-N72, illustrated in Figure A14 (top). Although the aliphatic backbone of rifampicin’s structure forms a macrocycle, the fact that it is mainly made up of single bonds renders it sufficiently flexible as to accommodate the conformational twists required for the formation of these interactions. In turn, intramolecular bonds reduce the number of atoms available for H bonding both as donors and acceptors, effectively bringing rifampicin closer to the respective thresholds of Lipinski’s rule of five [3,4,81].

Figure A14 (bottom) shows the average instant number per rifampicin of H bonds formed between rifampicin donor groups (from the aromatic rings, aliphatic chain, or piperazine ring) and POPC acceptor atoms, the latter being grouped into phosphate or ester O atoms. It is clear that the aromatic rings and aliphatic chain are mostly responsible for H bonding, mainly to the phosphate moiety. On average, each rifampicin molecule establishes 2.7 (*an*) and 3.4 (*zw*) H bonds with surrounding POPC phosphate groups. Together, they result in a very strong interaction, which is apparent in the RDFs of Figure 7 (bottom) and is probably responsible for the dragging of lipid headgroups upon rifampicin internalization, since they are observed for all rifampicin molecules, even the most internally located ones. Curiously, H bonding from the protonated piperazine N2 atom of *zw* to phosphate is infrequent. This is probably on account of steric hindrance, preventing effective bonding from this protonated tertiary amine group. In turn, the peaks in the RDFs of POPC P atoms around the piperazine ring (Figure 7, top) may now be interpreted as secondary to the H bonding from the aromatic ring phenol groups. This agrees with the lower values and lesser definition of RDFs of POPC P around the piperazine compared to those around the rings. Even though an electrostatic attraction between the protonated piperazine and the negatively charged phosphate may be present as commented above, this interaction is weaker than the multiple H bonds provided by the phenolic and hydroxyl groups.

#### 3.2.3. Rifampicin-Induced Bilayer Perturbation

From the data of Figure 5, namely, the reduced distance between the POPC P atoms and the center of the bilayer for lipids close to rifampicin molecules, one can infer that the latter induce significant local perturbation of the POPC membrane. From the preceding section, we can attribute this reduction to strong H bonding between rifampicin phenolic groups and lipid phosphate O atoms, which persists even for internalized rifampicin molecules. The latter end up dragging the phosphate groups of these nearby lipids toward the center of the bilayer.

**Figure 8:**
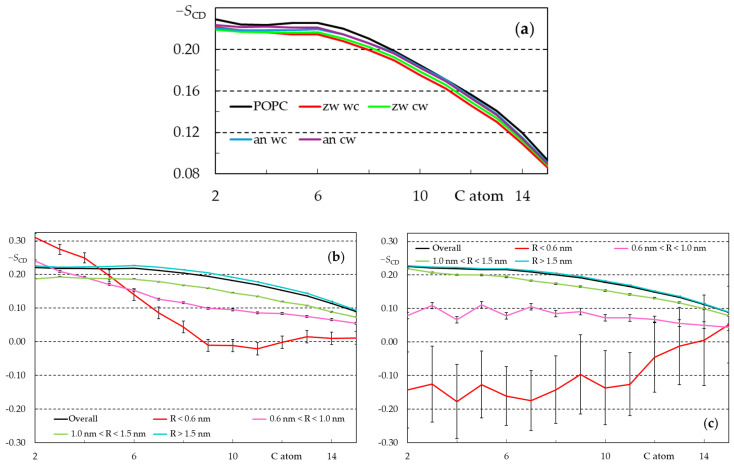
Calculated average deuterium order parameter (|*S*_CD_|) profiles for all *sn*-1 POPC acyl chains in the different simulations (**a**), and the last 200 ns of each simulation ((**b**), *an*, *w* simulation; (**c**) *zw*, *w* simulation) for different ranges of lateral distance R to the nearest rifampicin molecule inserted in the same leaflet.

**Figure A17:**
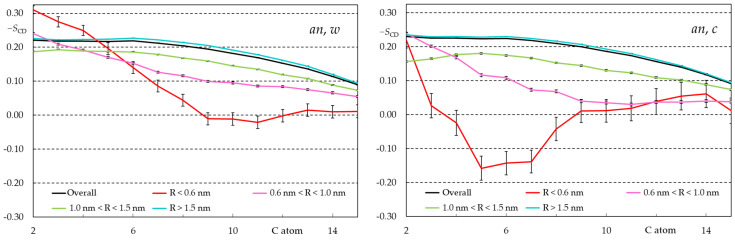

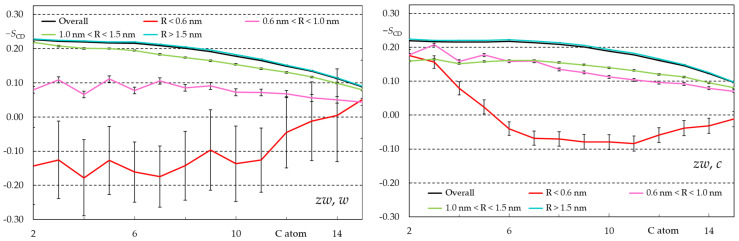
Calculated average deuterium order parameter (|*S*_CD_|) profiles for the last 200 ns of each simulation, for different ranges of lateral distance R to the nearest rifampicin molecule inserted in the same leaflet.

Even though this perturbation arises mainly from interactions with the lipid headgroup, its effects may spread to the hydrocarbon region of the bilayer. To check on this, deuterium order parameters (|*S*_CD_|) were calculated both for the *sn*-1 chains of all lipids in the simulations (Figure 8a) and for those situated at different ranges of distance to the closest rifampicin molecule (see Figure 8b,c for *w* simulations, Figure A17 for *c* simulations).

Figure 8 shows that interactions between rifampicin and POPC lipids cause an overall decrease in the deuterium order parameters of all carbon atoms along the chain. This decrease appears to be more pronounced in the *zw* than in the *an* species. For each ionization state, the overall perturbation is larger in the *w* simulations than in the *c* ones, in part because of the higher solute concentration in the former.

For a more complete characterization of these perturbations, the order parameters were also calculated for each individual *sn*-1 acyl chain and binned and averaged according to its distance R to the center of mass of the closest rifampicin molecule, in the same bilayer leaflet. As clearly visible in Figure 8b,c, the association of rifampicin with the membrane causes a very significant decrease in membrane order for both forms and all carbon positions along the chain (with the sole exception of a moderate increase in the order of the first carbon atoms of closest molecules for the *an*, *w* simulation, Figure 8b). For all systems, order parameters close to zero or even negative are calculated in the R < 0.6 nm range for at least some of the *sn*-1 chain carbon atoms, denoting a local lack of preferential orientation or predominance of orientations parallel to the membrane plane for those positions along the chain. While significant local perturbation has been reported for other biologically relevant molecules such as bile acids [82], the alterations shown in Figure 8b,c are more severe and, significantly, extend for longer distances to the solute center of mass (note that even lipids at R > 1.0 nm are clearly affected). The reasons for this are, at least, twofold. On the one hand, as described above, H bonding from multiple rifampicin donors to POPC phosphate atoms drags the headgroups of nearby lipids toward the center of the bilayer, inducing a local decrease in the bilayer thickness and, therefore, expected local disordering on a larger scale than that caused by solutes with fewer such interactions. On the other hand, the sheer size of the rifampicin molecule (~1.6 nm between the furthermost atoms) implies that this effect cannot be circumscribed to a limited region of the bilayer. In turn, this perturbation renders the bilayer more permeable, in accordance with the experimental results of Section 3.1.2. Interestingly, the more extensive membrane perturbation observed by rifampicin zwitterionic species and the expected stabilization of this species relative to the negatively charged one would favor rifampicin permeation through transient membrane perturbation in the case of negatively charged membranes. This would compensate for the lower affinity of rifampicin for lipid compositions representative of bacterial membranes [21], leading to an efficient internalization of rifampicin.

Our results of Section 3.2.2. show that while hydrogen bonding between membrane-inserted rifampicin and lipid atoms exists, it is probably diminished compared to water-solvated rifampicin. On the other hand, as shown in this section, significant perturbation of the membrane results from rifampicin insertion. Together, these two results agree with the experimental observation that rifampicin partition to POPC vesicles is driven by the change in entropy, rather than in enthalpy (which is actually unfavorable). It is also noteworthy that the rifampicin concentrations (1–2 mol%) used in the simulations do not exceed the expected local membrane concentrations in the experiments described in Section 3.1.1. The significant local perturbation of membrane order induced by rifampicin is also the likely explanation for the faster CBF permeation observed for an increasing rifampicin concentration (Section 3.1.2).

#### 3.2.4. Free-Energy Profiles from Umbrella Sampling MD

**Figure A18:**
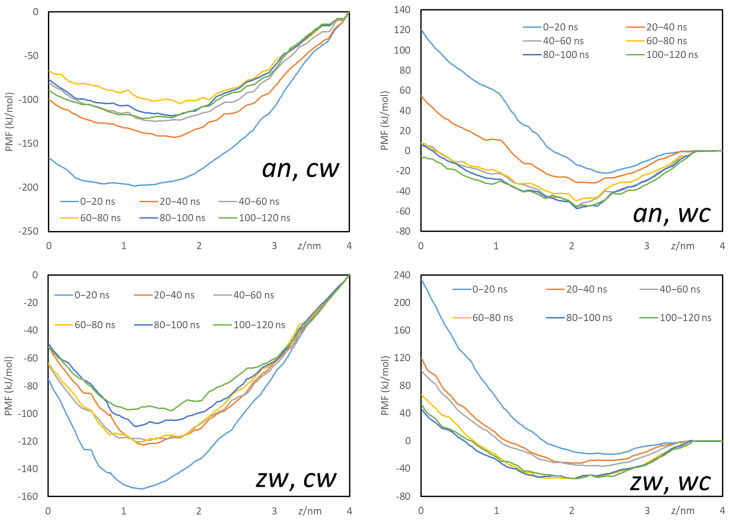
Variation in the calculated PMF profiles as a function of the distance between the rifampicin and local bilayer centers of mass *z* (using, as reference, the free energy at *z* = 4.0 nm), using 20 ns time ranges starting at increasing initial times (from *t* = 0, light blue, to *t* = 100 ns, green).

From US simulations, free-energy profiles were calculated for the two considered rifampicin forms, as described in detail in Section 2.2. The convergence of these profiles was assessed following the procedure of reference [83]. Figure A18 shows the variation in the PMF profiles obtained using different segments of the 0 < *t* < 120 ns simulation time range. For the *zw* species, it is visible that when the starting configurations for US are obtained by pulling molecules from the center of the bilayer towards the water medium (*cw* simulation), it was not possible to obtain converged PMF profiles (Figure A18). The *cw* pulling procedure leads to the establishment of H bonds between rifampicin molecules leaving the bilayer and headgroups of nearby POPC molecules, which are also pulled together with rifampicin molecules. This ultimately leads to a very large energy barrier for rifampicin desorption, failure to obtain a constant free-energy value even for large distances to the center of the bilayer, and (at least for *zw*) ultimately, non-converged PMF profiles. For the *an* species, although the profiles appear to converge for longer simulation times, the free-energy plateau in the water medium is still absent. Improved, even if possibly not definite, convergence is observed when the starting configurations are obtained by pulling molecules from the water medium into the center of the bilayer (*wc*). In this case, the main problem occurs when the pulled rifampicin molecule inserts into the bilayer. When it is pulled across the water/lipid interface, H bonds are formed with lipid headgroups, most of which are maintained when the rifampicin molecule is further pulled into the bilayer core. In both sets of simulations, the bilayer is significantly deformed after the pulling simulation, and these perturbations do not fully disappear even after 120 ns of US.

**Figure 9:**
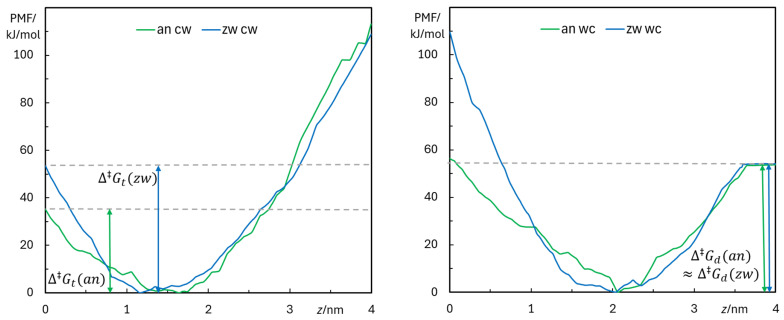
Free-energy profiles obtained for the *an* (green) and *zw* (blue) species, with molecules pulled in the *cw* (**left**) and *wc* (**right**) directions. The last 60 ns of the 120 ns sampling simulations were used. Free-energy barriers of translocation (${∆}^{‡}G_{t}$) and desorption (${∆}^{‡}G_{d}$), calculated from the *cw* and *wc* simulations, respectively, are illustrated in the corresponding panel.

**Figure A19:**
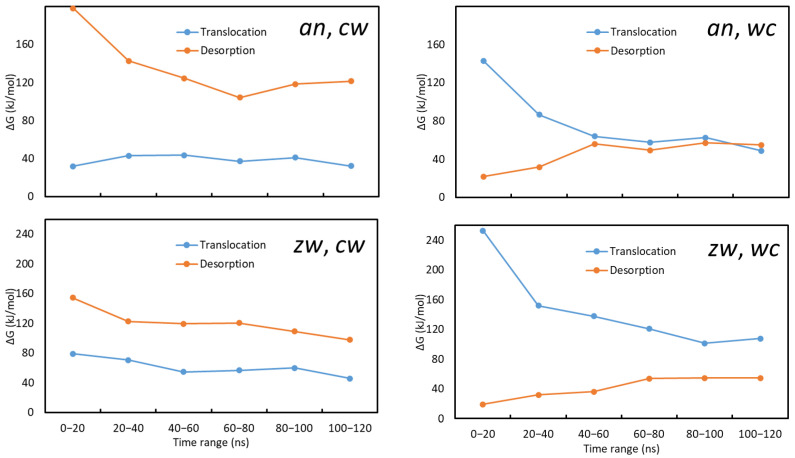
Free-energy barrier heights associated to the translocation (blue) and desorption (orange) processes, calculated using different 20 ns time ranges for sampling.

Another way to assess PMF convergence is to plot the increases in free energy from the minimum value to the maximum at the center of the bilayer, *z* = 0 (translocation barrier) or to the value in the water medium, *z* = 4.0 nm (desorption barrier). Figure A19 shows the variation in the two barriers for all scenarios, highlighting that in most cases systematic changes occur when considering the first segments of the US simulations. These variations tend to diminish when the starting times for sampling are increased. As a compromise between convergence and allowing a sufficiently ample time window for sampling, PMF profiles derived from using the last 60 ns of each restrained simulation are shown in Figure 9.

From the above discussion, it could be anticipated that pulling in the *wc* direction from water to the equilibrium location, and in the *cw* direction from *z* = 0 to the equilibrium location, would lead to situations with the least perturbation in terms of lipid molecules pulled together with rifampicin, especially allowing the initial 60 ns for equilibration and only using the final 60 ns for sampling. In accordance, these are the situations that mostly better reduce the systematic variations and approximate a plateau at long starting times in the energy barrier plots of Figure A19. Such a reasoning would lead to almost identical desorption energy barrier estimates, ${∆}^{‡}G_{d}=$ 54 kJ/mol for the two species, compatible with the existence of membrane-inserted rifampicin in both ionization states hinted at from the experiments of Section 3.1.1. Conversely, clearly different translocation energy barrier estimates ${∆}^{‡}G_{t}$ of 35 kJ/mol (*an*) and 53 kJ/mol (*zw*) are found. Because of the problems caused by the bilayer deformation during pulling, and consequent possibly incomplete convergence of the PMF profiles, these values cannot be considered as definite. Nevertheless, they can be interpreted considering the structure and charge distribution of the two rifampicin forms, as well as by comparison with previous reports. While the *zw* form of rifampicin has no net charge, it presents two oppositely charged, separate groups (an anionic phenolate and a cationic piperazinium). Therefore, it is actually reasonable that it is harder for it to translocate across the hydrocarbon core of the bilayer than it is for its *an* counterpart, which only bears the charged phenolate. In simple reductive terms, the translocation of *an* rifampicin may be viewed as controlled by that of a single negative charge, whereas that of *zw* involves the transport of two separate charges, implying additional free-energy cost. A similar behavior was reported by Magalhães et al. for the zwitterionic and cationic forms of rhodamine B [84]. In that study, the zwitterion displayed a free-energy barrier for translocation higher than that of the cation, with a difference between them of the same order as that observed here for the two forms of rifampicin.

## 4. Conclusions

Despite only verifying one (Clog *p* ≤ 5) and violating three of the four criteria of Lipinski’s rule for good oral bioavailability (MW > 500 g/mol, >5 H bond donor groups, and >10 H bond acceptor groups), the broad-spectrum antibiotic rifampicin demonstrates remarkable efficacy when administered orally. This study sheds light on the unexpectedly high bioavailability of rifampicin and similar bRO5 drugs through an in-depth investigation of rifampicin’s interaction with lipid membranes. A combination of experimental techniques—including isothermal titration calorimetry, absorption and fluorescence spectroscopy, dynamic light scattering (DLS), and zeta potential measurements—and computational molecular dynamics simulations provided unprecedented insights.

Using POPC bilayers as biomimetic models for biomembranes in general and those of mammalian cell membranes in particular, revealed distinct interactions not observed in prior studies employing non-polar solvents. Notably, rifampicin perturbs membrane barrier properties, inducing significant local disorder and enhancing CBF permeability by orders of magnitude, all without causing membrane disruption. This mechanism facilitates the permeation of large, polar molecules like rifampicin while minimizing cellular toxicity.

Another key finding is the stabilization of rifampicin’s negatively charged species, contrary to conventional expectations favoring its neutral form. Molecular dynamics simulations suggest that this stabilization arises from the deep insertion of the piperazine group into the membrane and the formation of intramolecular hydrogen bonds.

These findings not only advance our understanding of rifampicin’s bioavailability but also provide a broader framework for rationalizing how other large, polar drugs circumvent traditional bioavailability constraints. This knowledge can guide the development of next-generation therapeutics with enhanced pharmacokinetic profiles.

## Data Availability

The data presented in this study will be sent to interested researchers upon request to the corresponding authors.

## Appendix A. Complementary Experimental Data and Analysis

### Appendix A.1. Protons Exchanged with the Buffer Due to Rifampicin Association with the LUVs

The calorimetric enthalpy measured by ITC (${\Delta H}_{obs}^{0}$) reflects the changes in the molecular interactions established with the aqueous medium and the lipid membrane, but it also contains enthalpy variations due to additional processes that change when rifampicin moves from the aqueous medium to the membrane. One such process is eventual changes in rifampicin ionization. The protons released/captured by rifampicin (${\Delta nH}^{+}$) are captured/released by the pH buffer present in solution, and the heat evolved depends on the ionization enthalpy of the buffer (${\Delta H}_{ionization}^{0}$). The dependence on the ${\Delta H}_{obs}^{0}$ with ${\Delta H}_{ionization}^{0}$ of different buffers allows obtaining ${\Delta nH}^{+}$ and the intrinsic enthalpy variation ${\Delta H}_{P}^{0}$, shown in Equation (A1).

(A1) $${\Delta H}_{obs}^{0}={\Delta H}_{P}^{0}+{\Delta nH}^{+}\;{\times \Delta H}_{ionization}^{0}$$

### Appendix A.2. Changes in LUVs Zeta Potential Due to Association of Rifampicin

The association of rifampicin with the POPC LUVs leads to a variation in the LUVs zeta potential (ζ), which provides information regarding the overall charge of rifampicin. The LUVs ζ as a function of the total concentration of rifampicin is provided in Figure 3B, and the formalism followed for the quantitative analysis of the results is provided below.

The association of rifampicin with the LUVs imposes a surface charge in the membrane surface ($\sigma_{0}$) that may be calculated from Equation (A2) [23], and depends on the overall charge of rifampicin when associated with the membrane ($z_{Rif}^{M}$), on the molar fraction of rifampicin in the membrane ($n_{Rif}^{M}/\left(n_{Rif}^{M}+n_{L}^{M}\right)$), and on the membrane surface area ($s_{M}$), which depends on the cross-sectional area of the membrane components ($S_{i}^{M}$), $s_{M}=S_{Rif}^{M}\;n_{Rif}^{M}+{\;S_{L}^{M}\;n}_{L}^{M}$,

(A2) $$\sigma_{0}=e_{0}\frac{z_{Rif}^{M}{\;n}_{Rif}^{M}+z_{L}^{M}{\;n}_{L}^{M}}{S_{Rif}^{M}{\;n}_{Rif}^{M}+S_{L}^{M}{\;n}_{L}^{M}}$$

where $e_{0}$ is the electron charge, 1.6 × 10^−19^ C. In the analysis of the results, a cross-sectional area of 1.7 nm^2^ was considered for rifampicin [9], and of 0.64 nm^2^ for POPC [61].

The surface charge generates a surface potential in the membrane, which, for small surface potentials ($\left|\psi_{0}\right|<25\;mV$), is related with the surface charge density by Equation (A3):

(A3) $$\psi_{0}=\sigma_{0}\frac{\lambda_{D}}{\epsilon_{0}{\;\epsilon }_{r}}$$

where $\epsilon_{0}$ is the vacuum permittivity (8.85 × 10^−12^ F m^−1^), $\epsilon_{r}$ is the relative permittivity of water (80 at 20 °C), and $\lambda_{D}$ is the Debye length. For solutions containing monovalent electrolytes, the Debye length is given by Equation (A4):

(A4) $$\lambda_{D}=\sqrt{\frac{RT\epsilon_{0}{\;\epsilon }_{r}}{2F^{2}I}}$$

where R is the gas constant (8.314 J mol^−1^ K^−1^), T is the temperature in Kelvin degrees, F is the Faraday constant (96,485 C mol^−1^), and I is the ionic strength ($\frac{1}{2}\sum_{i}^{n}C_{i}z_{i}^{2}$) in Molar units, which corresponds to the salt concentration for monovalent salts.

The number of rifampicin molecules in the membrane, which is needed to calculate the surface charge density from Equation (A2), may be calculated from the partition coefficient, Equation (A5), where $\bar{V_{L}}$ is the molar volume of the lipids in the membrane (considered equal to 0.756 dm^3^ mol^−1^ [22]), and $K_{P}^{obs}$ is the partition coefficient observed at the experimental conditions followed.

(A5) $${\;n}_{Rif}^{M}={\;n}_{Rif}^{T}\frac{K_{P}^{obs}\bar{V_{L}}\left[L\right]}{1+K_{P}^{obs}\;\bar{V_{L}}\left[L\right]}$$

The observed partition coefficient depends on the membrane surface potential and on the overall charge of rifampicin, and it may be calculated from the intrinsic partition coefficient ($K_{P}$) using Equation (A6).

(A6) $$K_{P}^{obs}=K_{P}\;e^{-\frac{z_{Rif}^{M}\;F\;\psi_{0}}{RT}}$$

The zeta potential (ζ) measured experimentally allows an independent calculation of $\psi_{0}$ and, therefore, of the surface charge density, the convergence between the two estimates for the charge density allowing to obtain the intrinsic partition coefficient (if the overall charge is known) or the overall charge (if the intrinsic partition coefficient is known). In this work, it was assumed that the partition coefficient obtained at 10 μM rifampicin and high ionic strength corresponds to the intrinsic partition coefficient, and this formalism was used to obtain the overall charge of rifampicin when associated with the membrane.

The zeta potential (ζ) measured experimentally corresponds to the electrical potential at the slipping plane, which includes a solvent layer that moves with the charged particle. It is, therefore, necessary to calculate $\psi_{0}$ from ζ, which may be performed using Equation (A7) (which is an approximation valid for small surface potentials observed in this work [56]).

(A7) $$\psi_{x}=\psi_{0}e^{-\frac{x}{\lambda_{D}}}$$

The distance between the LUVs surface and the slipping plane (d) has been estimated as being 2 Å, allowing the calculation of the surface potential from the measured zeta potential, as shown in Equation (A8).

(A8) $$\psi_{0}=\zeta \;e^{\frac{d}{\lambda_{D}}}$$

The corresponding surface charge density may then be calculated from $\psi_{0}$ using Equation (A3). The combination of Equations (A3) and (A8) leads to Equation (A9), which allows the direct calculation of the surface charge density from the zeta potential measured experimentally.

(A9) $$\sigma_{0}=\zeta \;\frac{\epsilon_{0}{\;\epsilon }_{r}}{\lambda_{D}}e^{\frac{d}{\lambda_{D}}}$$

A note should be given to the units of the variables and parameters in the above equations, which must be SI for the direct use of the equations.

### Appendix A.3. Thermograms Obtained by ITC for the Addition of POPC LUVs to 100 μM Rifampicin

The association of rifampicin with POPC LUVs was characterized by ITC at different rifampicin concentrations and in the presence and absence of NaCl to evaluate the effects of ionic strength. A large decrease was observed in $K_{P}^{obs}$ with the increase in rifampicin concentration, which could not be justified by Equation (A6), and this was accompanied by variations in the interaction enthalpy. This behavior suggests a complex interaction between rifampicin and the POPC membrane at high rifampicin concentrations. In fact, at 100 μM rifampicin, the thermogram obtained cannot be described by a simple partition and shows the presence of endothermic and exothermic events (Figure A1).

### Appendix A.4. Effect of Rifampicin on the Rate of CBF Leakage

The variation in CBF fluorescence intensity over time when initially encapsulated at 50 mM inside POPC LUVs is shown in Figure A2, in the absence and presence of different concentrations of rifampicin. The fluorescence corresponding to full equilibration with the aqueous medium outside the LUVs was obtained after breakdown of the LUVs by the addition of triton X-100 to a final concentration of 1%. The fluorescence intensity observed is shown in plot A, and the % of CBF that leaches out of the LUVs is represented in Plot B. The latter was calculated from the intensity obtained at a given time ($I_{t}$) and in the presence of 1% triton X-100 ($I_{\infty }^{Tx}$), assuming a linear relation between the fluorescence intensity at a given time and the fraction of CBF outside the LUVs (Equation A10), which corresponds to the usual procedure when using this assay.

(A10) $${CBF}_{out}\left(\%\right)=100\;\frac{I_{t}-I_{0}}{I_{\infty }^{Tx}-I_{0}}$$

The lines correspond to the best fit of a bi-exponential function, with the corresponding rate constants being shown in plot C. The presence of rifampicin in the membrane imposes a negative charge at the membrane surface, which was expected to decrease the interaction of CBF with the membrane and, thus, slow down its permeation if occurring through a partition/diffusion mechanism [68]. A strong increase in the rate of CBF permeation is, however, observed, suggesting that rifampicin is compromising the membrane barrier properties.

Despite the clear overall trend, a closer inspection of the raw data shown in plot A points towards several possible problems with this methodology that may compromise a quantitative interpretation of the results. At the concentration of LUVs used in the assay, 0.06 mM POPC, the absorption of CBF at the excitation wavelength was much lower than 0.1, thus guaranteeing a linear relation between the fluorescence intensity and the concentration of CBF outside the LUVs. However, rifampicin absorption is significant at CBF excitation and emission wavelengths (Figure A3), leading to a significant inner filter effect and, thus, a decrease in the fluorescence intensity. Because the absorption of the solutions is maintained throughout the experiment, this effect should be kept constant and would not influence the fractional increase in CBF fluorescence due to leakage. Surprisingly, the effect of rifampicin on CBF fluorescence is not the same at *t*_0_ and after the addition of Triton X-100 (Figure A4). This points towards the presence of effects dependent on the proximity of rifampicin and CBF, which would depend on the extent of CBF leakage and, thus, influence the time variation of the fluorescence intensity. One possible effect is FRET between rifampicin associated with the LUVs and the encapsulated CBF. The Förster radius for FRET between CBF and rifampicin was calculated as being close to 3 nm (details in Appendix A.5). This shows that efficient FRET is not expected from CBF in the lumen of LUVs with a diameter of 100 nm. The higher quenching efficiency observed at *t*_0_ thus suggests that a significant fraction of CBF is associated with the lipid bilayer. The association of CBF with lipid bilayers was suggested in a recent publication for LUVs prepared from DPPC:DPPG:cholesterol 75:10:15, with an estimated partition coefficient of 2.9 × 10^4^ that increased up to 6.3 × 10^4^ in the presence of high concentrations of the cationic drug hydrochlorothiazide [85]. From the data reported in Figure A2, it is not possible to estimate the amount of CBF associated with the lipid membrane, namely because association with the mixed Lipid-Triton X micelles cannot be discarded. The reported partition coefficient seems, however, unrealistically large for a polar and charged molecule such as CBF, and would correspond to 100% of the encapsulated CBF being associated with the inner leaflet of the LUVs due to the very large lipid concentration in this compartment. A non-negligible association of CBF with the lipid bilayer may, however, occur, and would lead to artifacts in the time dependence of the fluorescence intensity.

### Appendix A.5. Spectral Overlap Between CBF and Rifampicin

The absorption spectra of CBF and rifampicin are presented in Figure A3, showing extensive overlap between rifampicin absorption and both the absorption and the fluorescence emission spectra of CBF, both contributing to significant inner filter effects if the total absorption is ≥0.1. The overlap between CBF emission and rifampicin absorption may also lead to fluorescence resonance energy transfer if the two molecules are in close proximity. The distance at which there is 50% FRET efficiency ($R_{0}$) is calculated from Equation (A11), with rifampicin molar absorptivity at each wavelength being calculated from the spectral shape and $\varepsilon_{Rif}$ = 1.5 × 10^4^ at 472 nm [12], CBF fluorescence quantum yield ($\phi_{F}^{CBF}\;$= 0.57) [79], the refractive index of water (n = 1.333) [86], donor-acceptor orientation in the dynamic isotropic limit [87], and the spectral overlap calculated from Equation (A12).

(A11) $$R_{0}=0.211{\left[k^{2}n^{-4}\phi_{F}^{CBF}\;J\left(\lambda \right)\right]}^{\frac{1}{6}}$$

(A12) $$J\left(\lambda \right)=\frac{\int_{0}^{\infty }F_{CBF}\left(\lambda \right)\;\varepsilon_{Rif}\left(\lambda \right)\;\lambda^{4}d\lambda }{\int_{0}^{\infty }F_{CBF}\left(\lambda \right)\;d\lambda }$$

The addition of rifampicin to LUVs containing encapsulated CBF leads to a decrease in CBF fluorescence even for very low absorptions at the excitation wavelength, indicating the importance of processes other than the trivial inner filter effect, namely, efficient FRET between CBF encapsulated and rifampicin associated with the LUVs. The fluorescence intensity at different rifampicin concentrations is shown in Figure A4.

Quenching of CBF encapsulated in the LUVs is much higher than after LUVs disruption (hollow vs. filled symbols), indicating the presence of local effects in addition to trivial inner filter effects. Decreasing the light pathlength leads to a decrease in the absorption and, therefore, on inner filter effects. Accordingly, no quenching is observed when the LUVs are disrupted by Triton X-100. However, CBF quenching in intact LUVs is maintained.

### Appendix A.6. Size of the POPC LUVs in the Absence and Presence of Rifampicin

The incubation of POPC LUVs with rifampicin at concentrations up to 80 μM does not lead to significant effects on the dynamic light scattering autocorrelation or in the corresponding average LUV diameter and polydispersity index (Figure A5).
